# Maternal transmission and limited virulent phage replication shape early infant gut virome maturation

**DOI:** 10.21203/rs.3.rs-11232673/v1

**Published:** 2026-10-05

**Authors:** Kim Kreuze, Lilia Björkgren, Christopher Turkington, Neda Nezam-Abadi, Sam Seymour, Andrey Shkoporov, Colin Hill, Ville-Petri Friman, Mikael Knip, Tommi Vatanen

**Affiliations:** 1 Institute of Biotechnology, Helsinki Institute of Life Science, University of Helsinki, Helsinki, Finland; 2 Department of Microbiology, Faculty of Agriculture and Forestry, University of Helsinki, Helsinki Finland; 3 School of Microbiology, University College Cork, Cork, Ireland; 4 APC Microbiome Ireland, University College Cork, Cork, Ireland; 5 Research Program for Clinical and Molecular Metabolism, Faculty of Medicine, University of Helsinki, Helsinki, Finland; 6 Center for Child Health Research, Tampere University and Tampere University Hospital, Tampere, Finland; 7 Turku Bioscience Centre, University of Turku and Åbo Akademi University, Turku, Finland; 8 Broad Institute of MIT and Harvard, Cambridge, MA, USA; 9 Liggins Institute, University of Auckland, Auckland, New Zealand

## Abstract

Early-life gut microbiome dynamics are increasingly recognized as important determinants of microbiome maturation, yet the ecological dynamics of its associated bacteriophages remain poorly resolved. Here, we performed bulk and virus-like particle (VLP)–enriched metagenomic sequencing and fecal metabolomic profiling of longitudinal fecal samples from 32 mother–infant dyads, incorporating an exogenous phage spike-in to enable absolute quantification of viral loads. Our results show that early infant gut phageome is defined by a profound lack of virulent phage genome copies while temperate phages remain relatively stable, challenging previous assumptions. We further identified extensive maternal sharedness of mainly virulent phages predicted to infect *Bacteroidaceae*, which could explain recovery of virulent phage absolute abundances. Virulent phage concentrations were further associated with several fecal metabolites including primary bile acids. These findings revise the current model of infant phageome assembly highlighting the underappreciated role of virulent phages.

## Introduction

While the infant gut microbiome is increasingly recognized as a key determinant of healthy development, the contribution of bacteriophages (phages) is yet understudied. Phages can alter bacterial abundances, diversity and community function through predation, lysogeny and horizontal gene transfer, suggesting they may be important drivers of microbiome assembly and infant health^1–5^. Thus, it is important to better understand how the infant gut phage community (phageome) is established during infancy.

Phageome assembly begins at birth alongside bacterial colonization. Although studies of infant gut phageome remain relatively scarce, a consistent observation is the apparent dominance of active temperate phages during early life compared to adulthood^6–11^. This has led to the current model that the pioneering phages in the infant gut originate from prophages that are being induced from the initially colonizing bacteria^7^. Evidence suggests that the abundance of active temperate phages decreases over the first year of life as the microbiome stabilizes and virulent phages become more abundant^9,11–13^. However, previous studies have largely relied on relative abundances, and it remains unclear whether apparent temperate-phage dominance reflects active expansion of temperate phages or scarcity of virulent phages in infancy. Absolute measurements of phage abundances are hence required to distinguish between these alternative explanations and to fully understand the ecological processes underlying phageome development.

Maternal transmission contributes to early microbiome assembly. For bacterial communities, mother-to-infant transmission plays a major role in microbiota establishment and infant development, as a substantial fraction of infant colonizing strains originate from maternal reservoirs and are implicated with infant health^14–18^. Transmitted taxa like *Bifidobacterium* and *Bacteroides* are thought to persist because they have adaptations that promote successful colonization of the infant gut, including efficient utilization of ingested substrates, surface adhesion and iron acquisition^18,19^. Similar ecological filtering may also shape phage transmission. Emerging evidence suggests that a subset of pioneering phages are maternally transmitted and can persist throughout infancy^9,17,20^. Several genomic and ecological features, including bacterial immune evasion, alternative codon usage and adaptations affecting host recognition have been proposed to facilitate persistence in the gut^20–22^. Although prophages have been proposed as a major source of maternally transmitted phages^9,23^, few studies have directly investigated the characteristics of maternally transmitted phages, and it is not entirely known how they affect phage community maturation.

Beyond regulating microbial community assembly, phage predation and lysogeny may also influence microbiome functions. For example, phage predation could select for bacteria that have acquired antiphage defense systems from mobile genetic elements^24–26^. Furthermore, phage infection can alter the physiology and metabolic activity of the bacterial hosts, which could result in measurable changes in gut metabolite profiles^4^. Indeed, experimental studies have demonstrated that temperate and virulent phages alter bile acid and other metabolic outputs *in vitro* and *in vivo*^4,27^. Metabolites such as bile acids are of particular interest as they are involved in complex bacterial metabolic pathways and important for host health^28,29^. However, the relationship between phage activity and gut metabolites has not been investigated in infants, leaving unclear how phage dynamics might contribute to functional maturation of the early-life gut metabolome.

In this study we combined absolute abundance virus-like particle (VLP) metagenomics, bulk metagenomics and metabolomic profiling of longitudinal samples from 32 mother-infant dyads to investigate phage population dynamics during early life. We show that infant phageome development is primarily driven by a lack of virulent phages rather than an excess of temperate phages as previously thought. Additionally, mother-infant sharing of virulent Bacteroidaceae phages helps explain the phage population recovery in infants. Finally, we find that virulent phages associate with primary bile acid metabolomics likely indirectly via their host bacteria. These results highlight the power of absolute abundances and provide a revised framework for understanding early phageome development, suggesting that virulent phages inherited from mothers could play a greater role in infant microbiome development than previously implied.

## Methods

### Cohort and DNA extractions

Maternal and infant fecal samples were collected in the EDIA study^30,31^. Inclusion criteria included informed consent signed by the parents and an eligible HLA genotype of the newborn conferring increased risk for type 1 diabetes. The study protocol was approved by the Ethical Committee of the Joint Municipal Authority of the Pirkanmaa Hospital District. VLP metagenome sequencing was performed on 167 fecal samples from 32 mother-infant dyads. Sequenced samples were taken from mothers at the time of delivery and from infants at 0.5, 1, 2, 3, and 6 months of age. The enrichment and sequencing were performed using a modified protocol reported previously with nine parallel negative controls for the enrichment^6^. Briefly, 100 mg of fecal sample was thawed on ice and homogenized in 1mL SM buffer. The slurry was centrifuged at 4,000 g at 4 °C for 30 minutes and the supernatant was filtered with a 0.45 μm PES filter (Sarstedt). This was followed by a second filtration step with a 0.45 μm Spin-X centrifuge tube (Merck) by centrifuging 13,000 g at 4 °C for 15 minutes. The 400 μl Filtrate was mixed with a *Lactococcus* phage Q33 (NC_049809.1) spike-in to a final concentration of 10^6^ PFU/ml. A spike in of the same concentration was added to SM buffer and processed in parallel as a negative control. To remove non-encapsulated nucleic acids, 8 U of TURBO DNase and 20 U of RNase I were added to the samples and incubated for 2 hours at 37 °C with intermittent mixing, followed by an incubation at 70 °C for 10 minutes. Next, particles were disrupted with proteinase K and nucleic acid was extracted with Qiagen Blood and Tissue Kit as manufacturer’s instructions. To detect RNA viruses, 11 microliters of template RNA/DNA elute was used for cDNA synthesis using SuperScript IV First Strand Synthesis System (Invitrogen/ThermoFisher) with random hexamer oligonucleotides as per manufacturer’s instructions. Nucleic acid samples were stored at −20 °C.

### Library preparation and sequencing

VLP DNA library preparation was performed in-house beginning with nucleic acid fragmentation using the M220 Focused-Ultrasonicator (Covaris) with peak power 50 W, duty factor 20 %, 200 cycles per burst, duration 35 seconds. Fragmented libraries were concentrated with Zymo Research Genomic DNA Clean & Concentrator columns. Libraries were prepared using the xGen^™^ ssDNA & Low-Input DNA Library Preparation Kit (IDT) as per manufacturer’s instructions, allowing us to amplify both single and double stranded DNA. Library quality and quantity was checked with the Agilent 4200 Tapestation and Qubit fluorometer respectively. Samples were sequenced by Azenta Life Sciences using the Illumina Novaseq X plus platform according to manufacturer’s instructions. Samples were sequenced with 2 × 150 bp paired-end reads configuration v1.5. Image analysis and base calling were conducted by the NovaSeq Control Software on the NovaSeq instrument. Raw sequence data (.bcl files) generated from Illumina NovaSeq was converted into fastq files and de-multiplexed using Illumina bcl2fastq program version 2.20. One mismatch was allowed for index sequence identification. All VLP samples were successfully sequenced.

### Viral operational taxonomic unit (vOTU) catalogue assembly

To identify viral operational taxonomic units (vOTUs) in our dataset, Illumina sequenced raw paired-end reads of samples and controls were inspected using FastQC (^32^ v. 0.11.5) and processed with a custom snakemake pipeline. Reads were first adapter and quality trimmed using trim galore (v.0.6.10,^33^, params: --paired –length 50) and seqtk (v.1.2-r94,^34^, params pair1: -e 10 -b 10, params pair2: -b 15). Next, Kneaddata (v. 0.12.0,^35^, params –trimmomatic-option MINLEN:70, --bypass-trf) was used to remove human and phiX174 DNA by mapping reads to the human genome database (hg37) and the reference phiX174 genome (NC_001422.1).

Quality and human filtered reads were next assembled into *de novo* assembled contigs using megahit (v1.2.9,^36^, default parameters). These resulting contigs were extended using COBRA (^37^, v1.2.3, params: cobra-meta -a megahit -mink 21 -maxk 141) with bowtie2 (^38^, v. 2.5.4) and coverm (^39^, 0.7.0, params: coverm contig --methods metabat). Contigs under 3 kbp were excluded from further analysis. These VLP assembled contigs and bulk assembled contigs from all samples of the EDIA cohort^31^ were analysed with a custom snakemake pipeline to identify viral contigs. Specifically, viral contigs were identified with a first run of geNomad (v1.7.4, database v1.7, params: default)^40^ followed by CheckV (v1.0.1, database v1.5, params: default)^41^ to quantify virus quality and identify prophages. The contigs that were identified as prophages with CheckV were submitted to a second pass of the pipeline. Contigs labelled as at least medium quality (i.e. completeness >50%) by CheckV were included in further analysis. In addition, we included VLP assembled contigs that were >10 kbp and had <=1 bacterial ORFs as being potentially viral. These viral contigs were next clustered with 773 196 viral and proviral sequences from MGV v2021.07.08, GPD v2020.09.02, ELGV v2023.12.11, Viral Refseq v10.1.2025, Prophage_DB v.7.18.2024 and the assembled viruses from an additional five studies^8,13,42–48^ by 95 % ANI over 85 % alignment fraction using CheckVs anicalc.py and aniclust.py scripts. The resulting 707 092 clusters were considered the vOTU catalogue with which we continued our analysis.

The catalogue was inspected for medium to complete quality representative sequences that were assembled from the EDIA cohort, excluding those that clustered with contigs assembled from negative controls, and their information is collected in table S1 according to the Minimum Information about an Uncultivated Virus Genome (MIUViG) standard^49^. The representative vOTUs that did not cluster with any viral sequences outside those assembled from the EDIA cohort were considered novel phage genome species.

### Annotation of vOTUs

To annotate vOTUs, the longest contig in the cluster was treated as a representative and annotated with an assortment of tools. The broad taxonomy of the vOTUs was determined by the taxonomy output of geNomad. The lifestyles of the vOTUs were first determined to be temperate if the representative sequence was annotated as a prophage by geNomad or CheckV, the remaining vOTUs were predicted to be either temperate or virulent using PhaTYP (^50^ version 3.2.2025, params: default) by inputting amino acid sequences predicted from geNomad. The host of each vOTU was predicted using iPHoP (^51^ v.1.3.3, params: --min_score 75) using a custom database that included iPHoP_db_Aug23_rw and all MAGs with a quality >50 % from the EDIA cohort assembled previously^31^. The custom database was built using gtdbtk de_novo_wf (2.7.2,^52^ params: --bacteria --outgroup_taxon p Patescibacteriota, --force) and iphop add_to_db. To simplify further analyses we retained only one predicted host assignment per vOTU, which was the best hit according to the prediction tools.

Viral auxiliary metabolic genes (AMGs) were identified by running the DRAM-v pipeline of DRAM (v1.4.6,^53^, params: default). Briefly, vOTUs were first processed using Virsorter2 (v.2.2.3, --seqname-suffix-off, --viral-gene-enrich-off, --provirus-off, --prep-for-dramv, --include-groups dsDNAphage,ssDNA, --min-length 1000, --min-score 0.5,^54^). Processed vOTUs were annotated and summarized with DRAM-v (DRAM-v annotate, DRAM-v distill, default parameters) assigning AMGs to each vOTU. Defense systems were identified using DefenseFinder 2.0.0 (params: default,^55–57^) with CasFinder 3.1.0 and defense-finder 2.0.2 models and Padloc v2.0.0 (params: default,^58^) with padloc database release v2.0.0. Defense systems labelled as phage defense candidate (PDC) and Hma-embedded candidate (HEC) were excluded to only include validated systems. Padloc output was processed to match and align to defense finder using code from a previous study^59^ and outputs were manually combined and dereplicated. In the case of contradictory overlaps from both tools, the output of defense finder was used. Antidefense systems were identified with the antidefense finder extension of DefenseFinder.

### Bacteria and functional abundance estimation

Processed bulk metagenome sequencing reads that were from the same samples with VLPs extraction were obtained from SRA under bioproject PRJNA821542. Bacterial species genome bin (SGB) relative abundance was quantified using MetaPhlAn4 (v 4.1.1,^60^, params: default) with mpa_vJun23_CHOCOPhlAnSGB_202403 database. Abundance of microbial functional potential was estimated using HUMAnN 3.0 (v.3.9,^61^). The functions were regrouped into Clusters of Orthologous Genes (COG) and Non-supervised Orthologous Groups (NOG) terms with the human_regroup_table command and normalized to relative abundance with humann_renorm_table.

### vOTU abundance estimation

To estimate the relative and absolute abundances of vOTUs in each sample, we first mapped cleaned VLP reads to the catalogue of 707 092 vOTUs with bowtie2. Contigs with a coverage breadth of at least 75% were considered present and reads per kilobase million (rpkm) were calculated for each using coverm (params: --min-covered-fraction 75 --methods rpkm) revealing 3419 vOTUs. vOTUs that were also present in any of the negative control samples were considered contaminants and removed from further analysis leaving 3272 vOTUs present. After removal of contaminants, rpkm values were used to calculate vOTU relative abundances. We used the relative abundance of *Lactococcus* phage Q33, which was present in the vOTU catalogue, and had known PFU/ml allowing us to calculate the absolute viral abundances in each sample and for each vOTU. The calculated absolute abundances were all log10 transformed for visualization and further analyses. All dilutions were taken into consideration in calculations. Five infant samples did not pass quality assessment and were left out of all analyses.

### Microbial diversity and abundance analyses

Shannon diversity was calculated for vOTUs and bacterial SGBs with vegan package (2.7–5,^62^). Associations with Shannon diversity and richness were assessed with GLMMs using the glmmTMB (v. 1.1.14,^63^) package with Tweedie and generalized poisson distributions respectively. Including all samples, alpha diversity metrics were modelled with mother-infant identity and read depth (viral models include DNA concentration) as covariates and family ID as a random effect. Using only infant samples, alpha diversity metrics were modelled with infant age in months, exclusive breastfeeding and read depth (viral models include DNA concentration) as covariates with family ID as a random effect. Associations were assessed using the lmerTest package (v. 3.2–1,^64^).

To overcome issues with extremely low vOTU prevalence, vOTUs were grouped by bacterial families they are predicted to infect (phage host families, PHF^65^). For fair comparison, bacterial SGBs were also grouped by bacterial families. PHF prevalences were investigated to see if they were associated with infant or maternal samples using GLMMs with a binomial distribution. The models had the same covariates and random effects as the alpha diversity models described above. NMDS ordination of Bray-Curtis dissimilarity matrices were calculated for bacterial families and PHF abundances using vegdist and metaMDS functions from vegan. Association between maternal and infant samples, infant age and NMDS coordinates on the x and y axes was assessed using LMMs using the lme4 package (v2.0–6,^66^) as described above without the read depth or exclusive breastfeeding as covariates. Bray-Curtis dissimilarity values for each mother-infant pair in each timepoint was used to see if dissimilarity changed over time. This was done with LMMs where infant age was a covariate and family ID the random effect. Associations between bacterial family and their corresponding PHF relative abundances in infants were investigated with GLMMs using ordered beta regression. Specifically, bacterial family associations with their corresponding PHFs were investigated with infant age and exclusive breastfeeding as covariates and family ID as random effect. PHFs and bacterial families with a prevalence <20 were not included in these models.

To model difference in total VLP absolute abundance between mothers and infants and over infant age, we built LMMs with the same covariates and random effects as with Shannon diversity analyses. Temperate and Virulent absolute abundances were compared between mothers and infants at each timepoint using paired Wilcoxon test. Longitudinal change in Temperate and Virulent absolute abundance in infants was done with LMMs as with Shannon diversity analyses.

Associations with PHF absolute abundances were assessed for virulent and temperate phages separately. This was done by running multiple LMMs using MaAsLin3 (v.00.99.16, (Nickols *et al*. 2026) params: normalization NONE, median_comparison_abundance FALSE, max_significance 0.25) with infant age, exclusive breastfeeding and gender as covariates and family ID as random effect. Associations with bacterial family’s relative abundance was done with the same covariates but with default MaAsLin3 parameters.

Bacterial and viral diversity and relative abundance associations with virulent phage content were explored using GLMM and LMMs respectively with infant age, exclusive breastfeeding, gender and virulent phage content as covariates and family ID as a random effect. The GLMM was built with a tweedie distribution and the LMMs were run with default MaAsLin3 parameters using bacterial SGB relative abundances. P_FDR_>=0.25 was considered significant for exploration analysis.

### Prophage induction analysis

Bulk metagenome assembled contigs that were predicted to contain a prophage by geNomad or checkV and were medium to complete quality (by checkV) were used for induction analysis. This was done by mapping bulk metagenome reads back to the contigs and using the genome coordinates of the prophages with PropagAtE (^67^ v1.1.0, default parameters). The outputted mean coverage of each prophage region was natural log transformed and subtracted by the natural log transformed mean coverage of the background contig, giving us the log transformed coverage difference (logRi). The effect of infancy on logRi was assessed using an LMM that had maternal-infant identity as a covariate and sample ID and family ID as random effects. We did not perform statistical tests with prophages that were labeled as active by PropagAtE (i.e. prophage-host coverage ratio >2) as only 24 prophages passed this threshold from 22 samples.

### Phage sharedness analysis

We counted a vOTU as being shared if it had a relative abundance exceeding zero in the mother and their corresponding infant at any timepoint. To include possible dormant prophages, we further mapped the clean bulk metagenome reads to the 3272 vOTUs and once again considered them present if they had a coverage breadth over 75 % using coverm. A permutation analysis (n=1000) was performed to see that the amount of vOTU sharing events was not random. To further get the proportion of shared active vOTUs we counted how many of the vOTUs that were present with VLP reads in infants were also detected in their corresponding mothers with VLP reads or bulk reads. This would account for vOTUs that were dormant in mothers and became active in infants or were active in both mother and infant. We incorporated the proportion of shared virulent and temperate vOTUs as an additional covariate in the virulent and temperate absolute abundance LMM models mentioned in the previous section to see if sharedness influenced abundance.

### Metabolomic analysis

Metabolomic data generated by liquid chromatography - mass spectrometry (LC-MS), was retrieved from^31^. A wide screen using all metabolites was used to assess whether fecal metabolites were affected by infant total VLP, temperate and virulent absolute abundance using multiple LMMs with MaAsLin 2^68^ (v.1.15.1, default parameters, R version 4.4.2). For these models the same covariates and random effects as the MaAsLin3 analysis mentioned previously were used, with the addition of the absolute abundance that was tested as another covariate.

Primary bile acids identified by the C18 neg method mentioned in^31^ were the main focus for the following analyses. The four primary bile acids (glycocholate, taurocholate, glycochenodeoxycholate and taurochenodeoxycholate) used to assess if specific PHFs and bacterial families affected their concentrations. This was done by running multiple LMMs with the same parameters as the previous models but replacing the lifestyle stratified absolute abundances with PHF absolute abundances (with prevalence exceeding 20 %) and centered log-ratio (CLR) transformed bacterial family relative abundances with a pseudocount of 10^−8^ (same families as the PHFs). All associations were FDR adjusted using the Benjamini-Hochberg procedure. To test whether the effect of PHF abundance on the primary bile acids was mediated by their host abundance, a mediation analysis was performed using piecewiseSEM^69^ (v.2.3.1). Both CLR transformed bacterial family relative abundances and PHF absolute abundances were used. The relationship between mediator (bacterial family) and predictor (PHF) was modelled with an LMM with infant age as a covariate and family ID as a random effect. The full model was an LMM with bacterial family, PHF and infant age as covariates and family ID as random effect. Total, direct and indirect effects with 95% confidence intervals were estimated using 1000 non-parametric resampling iterations with semEff^70^ (v.0.7.2, params: R=1000).

HUMAnN quantified functions were regrouped into KO groups with the human_regroup_table command and normalized to relative abundance with humann_renorm_table, which was further log10 transformed. The log10 transformed relative abundances of bile salt hydrolase (BSH, K01442) and its *Bifidobacterium longum* stratified abundances were used to assess their effects on primary bile acid concentrations. This was done with LMMs with infant age, exclusive breastfeeding and chololylglycine hydrolase abundance as the covariates and family ID as the random effect.

### Functional potential analyses

Bimodality of the virulent phage relative abundances in each infant timepoint was tested using Hartigans dip test for multimodality using the package fitdistrplus v.1.2–4. Infants with cumulative virulent phage relative abundance >50% and <=50% were labeled with the categorical variables high virulent phage content and low virulent phage content respectively. The COG functions were tested for association with virulent phage content by running multiple LMMs using MaAsLin2. The models had virulent phage content, exclusive breastfeeding, infant age and gender as covariates and family ID as random effects. The non-stratified COG functions that associated with phage virulence level and had an P_FDR_<0.25 were considered significant. To narrow down on functions related to phages, mobile genetic elements, phage defense and antimicrobial resistance, descriptions with the strings “mobile, transpos, integr, plasmid, recomb, vir, symbio, phag, conju, resist, defen, tox, crisp, tail, RM, Restric, caps, head, base, sheath and tape” were used to choose the significant COG categories to visualize.

Bulk metagenome assembled contigs were annotated with DefenseFinder as previously described. The number of defense systems were tallied for each infant sample to estimate defense system richness. Bacterial defense system richness association with temperate phage abundance was assessed with two separate GLMMs with generalized poisson distribution. Both had infant age, log transformed bulk reads, exclusive breastfeeding and gender as covariates and family ID as random effect. One had virulence level and the other log transformed absolute temperate abundance as covariates.

### Statistical analyses

All statistical analyses were performed in R version 4.6.1 (2026-06-24 ucrt) unless otherwise stated. Linear mixed models were performed for lme4 v2.0–6. Generalized linear mixed models were executed with glmmTMB v.1.1.14. Significance of covariates was assessed using lmerTest v.3.2–1 and model validity was investigated by plotting residuals.

### Data visualization

All plots were produced in R version 4.6.1 (2026-06-24 ucrt) with ggplot2 v. 4.0.3^71^.

## Results

### Maternal and infant vOTU characteristics were predominantly distinct

We investigated the virus like particle (VLP) enriched and bulk metagenomes of longitudinal fecal samples from 32 mother infant dyads from the EDIA cohort to characterize the actively replicating and dormant fecal viral populations in infants and their mothers (Fig. 1A). All the infants were breastfed, four were born by c-section and three were administered antibiotics at some point. Clustering of *de novo* assembled contigs from VLP metagenomes and viral genomes from several databases revealed 1445 species-level virus operational taxonomic units (vOTUs; medium to complete quality by checkV) after contamination removal within the EDIA cohort (Table S1, Fig S1). Of these, 453 (31.3%) vOTUs were not found in existing databases and were thus novel. While the sequencing of VLP samples included a reverse transcription step, we only detected 12 RNA vOTUs that were plant, human and fungal viruses (orders *Martellesvirales*, *Durnavirales*, *Patatavirales*, *Tymovirales* and *Picornavirales*). We additionally identified only five DNA vOTUs that infect humans (families *Adenoviridae*, *Anelloviridae* and *Circoviridae*) and two that could not be identified by geNomad. The remaining vOTUs were all bacteriophages with the majority within the class *Caudoviricetes* (realm *Duplodnaviria*) and the family *Microviridae* (realm *Monodnaviria)*. Notably, most of the *Microviridae* vOTUs were assembled from maternal samples, suggesting that these phages were predominantly maternal and less common in infants.

By further mapping the VLP enriched reads to the *de novo* assembled genomes and a catalogue of approximately 700 000 vOTUs, excluding those that mapped to our negative controls, we identified 3272 actively replicating vOTUs present in our cohort (Fig. 1B). Slight majority of these vOTUs were predicted to be virulent (51.8 %) although they were not evenly distributed between the hosts they were predicted to infect. For example, most of *Caudoviricetes* phages infecting *Bacteroidaceae* were classified as virulent (66.2 %, Fig. 1C). On the other hand, most vOTUs belonging to *Caudoviricetes* infecting *Lachnospiraceae* and *Clostridiaceae* were predominantly temperate (69.3 % and 84 % respectively). Different phage types were more common in mothers or infants. For example, *Microviridae* phages were almost exclusively found in maternal samples and predicted to mostly infect *Bacteroidaceae*. Further, *Caudoviricetes* phages predicted to infect *Bifidobacteriaceae*, *Clostridiaceae*, *Enterobacteriaceae* and *Enterococcaceae* were more prevalent in infant samples (GLMM, p<0.05).

These data show the majority of the vOTUs in human gut microbiomes were *Caudoviricetes* and *Microviridae* but were not evenly distributed by lifestyle or infected host predictions. Additionally, we note that vOTUs seem to be mostly restricted to being in either mothers or infants with only a small number being present in both (3.9 % of vOTUs). To understand how these viral dynamics change between mothers and infants, we next explored viral abundance changes between mothers and their infants.

### Highly personal viromes mirror bacteriome structure

To explore temporal phage-bacteria dynamics, we compared viral and bacterial relative abundances in mothers and infants. As expected, the total viral and bacterial richness and Shannon diversity indices were significantly higher in mothers compared to infants (GLMM, p<0.001). Additionally, we observed an increase in bacterial but not viral Shannon diversity in infants over time (Fig. 2A, B, GLMM, β=0.045, p=0.002). Furthermore, bacterial and phage Shannon diversities were not correlated in mothers nor infants. There were also no differences in viral diversity metrics according to any of our infant metadata even though we did detect lower bacterial richness in exclusively breastfed infants (GLMM, β=−0.169, p=0.047).

Infant viromes were highly variable and personal with the vast majority of vOTUs being singletons and the most prevalent vOTU was present in only 20% of the samples (Fig. S2A, B). Thus, to compare bacterial and viral population structures, we compared abundances of bacterial families and vOTUs based on their predicted host family (PHF). Non-metric Multidimensional Scaling (NMDS) analyses of the Bray-Curtis dissimilarity of bacterial families and PHF population structures revealed similar patterns (Fig. 2C, D). Specifically, the mothers clearly separated from their offspring by both the PHF and bacterial family composition (LMM, p<0.001). In addition, we found that both bacterial families (LMM, p<0.001) and PHF (LMM, p<0.001) significantly developed toward maternal profiles over time. This was further supported by the bacterial and viral Bray-Curtis dissimilarities between mothers and infants significantly decreasing with infant age (LMM, β=−0.018, p<0.001 and β=−0.014, p=0.049, Fig. S3A, B). Several bacterial families and PHF relative abundances had statistically significant positive correlations across all infant samples (Fig. 2E, F, Table S2), indicating that phage and bacterial structures mirrored each other. These results show that while infant viromes were highly personal, clustering phages based on predicted hosts reveals how phage and bacterial population structures reflect each other.

### Virulent, not temperate, phage dynamics define early infancy phageome assembly

By factoring in the phage spike-in in the sequencing protocol, we estimated the total VLP genome copies per gram of feces as a measure of absolute abundance. Leveraging this, we found that maternal samples had a median of 4.89 × 10^8^ VLP genome copies per gram, which is within the range of previous reports^6,72,73^. On the other hand, maternal viral loads were significantly higher compared to infants (LMM, β=0.739, p<0.001, Fig. S4), with a median of 3.05 × 10^7^ genome copies per gram. Additionally, VLP concentrations gradually increased with infant age (LMM, β=0.075, p=0.009) from a median of 1.22 × 10^7^ genome copies per gram in two-week-old infants to a median of 8.76 × 10^7^ in six-month-old infants. These results suggest that the infant virome concentrations do not yet reach stable adult levels by the age of six months.

We further investigated the VLP genome copies of temperate and virulent phages in relation to the virome development. Cumulative relative abundances of temperate phages were higher in infants compared to mothers, as observed in previous studies (Fig. S5). However, there was no statistically significant difference in temperate phage absolute abundance between mothers and infants when all infant timepoints were pooled together (LMM, p=0.812). Cross-sectionally, there was a small difference in temperate phage concentrations between the mothers and infants at timepoints 0.5 and 1 (Paired Wilcoxon, p<0.05, Fig. 3A), having medians of 2.53 × 10^7^, 8.65 × 10^6^ and 6.37 × 10^6^ genome copies per gram respectively. This effect disappeared by month 2 (Paired Wilcoxon, p=0.090). On the other hand, virulent phage concentrations were significantly higher in mothers compared to infants across all timepoints (Paired Wilcoxon, p<0.001, Fig. 3B), with mothers and infants having a median of 4.66 × 10^8^ and 7.11 × 10^6^ genome copies per gram, respectively. Additionally, while both virulent and temperate phages significantly increased in concentration with infant age, the increase was more prominent with virulent phages (LMM, β=0.187, p=0.001 and β=0.079, p=0.045). This data indicates that infant gut phageome development is characterized by a lack of virulent phages, rather than an increase in temperate phage abundances.

We next investigated differences in prophages induction between mothers and infants. This was achieved by inspecting log transformed coverage differences (logRi) between each medium to high-quality prophage and its background contig. We found that there was no significant difference in logRi between mothers and infants (LMM, p=0.809, Fig. 3C). Additionally, only 24/705 prophage (3.4 %) passed the threshold to be considered significantly induced, suggesting that induction was not common throughout the cohort. However, the prevalence of induced prophages was higher in infancy given that 23 of these prophages were significantly induced in 16 out of the 32 infants in this study. This suggests that induction occurred at low overall frequencies in both mothers and infants.

Finally, we investigated the characteristics of the temperate and virulent PHFs whose abundance changed over time. We found that the increases in virulent phage copies along with infant age were driven by *Bacteroidaceae* phages (LMM, P_FDR_<0.25, Fig. 3D), which were predominantly unknown Caudoviricetes. While *Microviridae* phages did contribute to this increase in *Bacteroidacea* phage abundance, they were only present in 6/29 infants by the sixth month. On the other hand, temperate *Lachnospiraceae*, *Oscillospiraceae* and *Lactobacillaceae* phage prevalences increased with infant age (GLMM, P_FDR_<0.25, Fig. 3D). Interestingly, these patterns were not reflected in the bacterial population as *Bacteroidaceae* and *Lactobacillaceae* did not significantly change over time (GLMM, P_FDR_<0.25, Fig. 3D). These results suggest that there are specific phage groups that increase in concentration in relation to infant age and that they do not necessarily follow their host abundance patterns.

### Maternal transmission is dominated by virulent phages

To observe if phage vertical transmission from mothers to infants or prevalence could help explain the lytic and temperate phage abundance in infants, we analysed the identity of vOTUs that were present in mothers and their infants across the whole data. This revealed that only 58 out of 3272 vOTUs were shared within 19 of the 32 mother-infant pairs. To include uninduced prophages to this comparison, we compared the shared vOTUs between mothers and infants using bulk metagenome reads. We found that 390 vOTUs were shared between 31 mother-infant pairs for 1154 times across all time points and a further permutation analysis verified that this sharing was not random (P <0.001, Fig. S6). Interestingly, despite most of the shared vOTUs being detected from the bulk metagenome data, the most shared vOTUs were virulent *Caudoviricetes* phages predicted to infect *Bacteroidaceae* (34.6% of shared vOTUs, Fig. 4A).

The composition of the shared vOTUs did not change over time, with *Bacteroidaceae* infecting virulent *Caudoviricetes* phages being the most shared across all timepoints (Fig. S7). Given this temporal stability, we next investigated whether maternal sharedness influenced infant viral loads. To do this, we focused on actively replicating infant vOTUs that were shared with mothers. The proportion of shared virulent vOTUs had a strong positive correlation with virulent VLP concentration in infants (LMM, β=0.788, p=0.004). We also observed an interaction with infant age, where the association between proportion of shared virulent vOTUs and virulent VLP concentrations was stronger at three and six months of age (LMM, β=0.225, p=0.039, Fig. 4B). In contrast, the proportion of shared temperate phages and temperate VLP concentrations were negatively correlated (LMM, β=−0.666, p=0.022, Fig. 4C). Together, these results suggest that virulent phages were more often shared between mothers and infants than temperate phages and that the proportion of shared phages correlates with infant absolute phage abundance with different patterns.

### Inverse relationship between phage replication and primary bile acids

Given that phage lytic replication can impact microbiome metabolic pathways, we next explored if there were correlations between phage absolute abundances and fecal metabolites. We found 2075 fecal metabolomic features that correlated with total phage absolute abundances (LMM, P_FDR_<0.25, Table S3), although only five were annotated metabolites. Exploring lifestyle specific associations, we ran the same model using virulent and temperate absolute abundances and found 1843 and 900 fecal metabolite hits with seven and one being annotated, respectively (Table S3). Interestingly, virulent but not temperate phage absolute abundances were inversely correlated with primary bile acids glycocholate and taurodeoxycholate (Fig 5A, LMM, P_FDR_<0.25). Since gut bacteria convert primary bile acids into secondary bile acids through bile salt hydrolase (BSH) activity, we hypothesized that phage-associated variation in primary bile acid could be mediated by BSH AMGs. However, no phage encoded BSH homologs were detected. In contrast, the abundance of BSH gene in the bulk metagenome was inversely correlated with all tested primary bile acids (LMM, p<0.001, Fig 5B). Investigation of the SGB stratification by carrier bacteria revealed that *Bifidobacterium longum* BSH gene was the most prevalent across the cohort and negatively correlated with the primary bile acids (LMM, p<0.001, Fig 5C).

As phage-encoded BSH could not explain the observed associations, we next asked whether the relationship with primary bile acids was driven by specific PHFs. Indeed, we found that *Clostridiaceae* phage absolute abundance and prevalence were positively associated with several primary bile acids, while *Bifidobacteriacea* and *Lactobacillaceae* phages were inversely associated with them (LMM, P_FDR_<0.25, Fig 5D). These patterns were largely mirrored by the corresponding bacterial hosts. Specifically, *Bifidobacteriaceae* and *Clostridiaceae* CLR transformed relative abundances and prevalences matched the direction of the phage associations with primary bile acids (LMM, P_FDR_<0.25, Fig 5E). While phages could be influencing primary bile acid concentrations by infecting BSH encoding taxa like *Bifidobacterium longum*, it could also be that the phages reflect host abundances. Indeed, mediation analyses suggested that bile acid associations in *Clostridiaceae* and *Bifidobacteriaceae* were completely mediated by the respective predicted hosts of the phages (Table S4). We therefore suspect these phage-bile acid associations are not biologically direct but instead arise from covariance with hosts involved in bile acid metabolism.

### Phage lifestyle and abundance implicated in community functions

Finally, we aimed to understand how virulent phages contribute to microbiome maturation since their concentration changed the most as infants aged. To this end, we leveraged the fact that in our dataset the relative abundances of virulent phages followed a bimodal distribution in most timepoints (Hartigans’ dip test, p<0.05, Fig. 6A). Thus, we defined samples as having either “high” (>50%) or “low” (<=50%) virulent phage content based on relative abundances in the VLP fraction. Samples with low virulent phage content had a lower total VLP concentration (LMM, β=−0.292, p=0.004), concordant with our previous observation that virulent phages were more abundant than temperate phages. Total phage Shannon diversity was lower in high virulent content group (GLMM, β=0.277, p=0.016). Bacterial diversities did not differ between the groups. Bacterial abundances had only statistically weak differences between the groups (Table S5). These results suggest that differences in phage lifestyles in infants do not appear to be strongly associated with changes in bacterial community structure.

We next tested for differences in bulk metagenome functional potential, estimated by HUMAnN3, between low and high virulent phage groups. We found 3195 COGs and ENOGs with statistically significant abundance differences between the groups (LMM, P_FDR_<0.25, Fig. 6B). Focusing on functional terms related to phages, mobile genetic elements (MGEs), antimicrobial resistance genes and phage defense systems, we found that these functions were more abundant in infants with low virulent phage activity (LMM, P_FDR_<0.25, Fig. 6C). These results could reflect increased abundance of lysogens, inducing prophages or both. Supporting this, we found that temperate, not virulent phages encoded more phage defense genes across most PHFs (Fig. 6D). Hence, we checked if defense system richness directly predicted from our bulk metagenome assemblies correlated with temperate phage abundances. While we found that defense system richness increased with infant age (GLMM, β=0.073, p<0.001), there was no evidence that it correlated with temperate or virulent phage abundances (GLMM, p>0.05). Together, phage lifestyle and abundances were associated with community functional potential, emphasizing the necessity of integrating phage profiling into microbiome studies.

## Discussion

In this study, we provide a revised model of infant gut virome assembly by employing absolute viral quantification across the first six months of life. Contrary to the established paradigm that early infancy is defined by a peak in temperate phage replication, our data demonstrates that the concentration of active temperate phages in infants is at first lower compared to mothers but is already comparable to maternal levels by two months of age. We show that the perceived dominance of temperate phages in early life is an artifact of relative abundance data, arising from a profound scarcity of virulent phages in the neonatal gut. We found that the maturation of the virome is primarily driven by the expansion of virulent phage populations, particularly those targeting *Bacteroidaceae*, which are significantly shaped by maternal transmission. Furthermore, we demonstrate that viral absolute abundances were implicated in faecal metabolite levels, including primary bile acids, possibly by mirroring bacterial hosts that deconjugate them into secondary bile acids. Finally, our results suggest that successful lysogen colonization may limit the niche space available for virulent phage expansion. Together, these findings highlight that the early life virome is not primarily defined by large induction events but rather by the dynamic acquisition of maternally derived virulent phages.

Our observed maternal VLP genome counts were comparable to existing data from adults. However, the observed infant VLP genome counts and their increase throughout infancy were lower compared to a previous study that calculated 1.6×10^9^ VLPs per gram in one month old infants by fluorescence microscopy^7^. While there could be both technical and biological reasons for differences between studies, differences in study design and the lack of maternal samples in their study further complicates comparisons between studies. One important technical difference is that our method identified and quantifies viruses through sequencing and bioinformatic while the Liang *et al*. counted DNA containing small particles, which include non-viral particles^74^. In contrast, it could also be that our genome copy concentration in mothers is an underestimate given that mothers have higher abundance of *Microviridae* phages and thus could bring the average genome size of phages in their samples below that of the Q33 spike-in.

With the VLP spike in, we obtained a more precise estimate of changes in concentrations of phages with different lifestyles and gained more biological insight into the dynamics of the infant gut virome. Specifically, we were able to show that temperate phage VLP concentrations dropped slightly in early infancy, but the concentrations recovered quickly to maternal levels. Conversely virulent phage VLP concentrations dropped more prominently, and although steadily increased with infant age, did not yet reach maternal levels by the end of sampling period. This accounts for previous observations where active temperate phages are at higher relative abundances in early infants compared to adults and decrease with infant age^9,11,75^. These findings could be potentially explained by a transmission bottleneck, where the transfer of a small fraction of maternal phages leads to a decrease of genetic diversity and population size in infants. Furthermore, these results indicate that virulent phages may be relatively more susceptible to such transmission bottlenecks compared to temperate phages. This could be explained by a relatively low chance of a free virion encountering a host in the newly colonized gut while prophages enter the new environment with their host allowing immediate replication or survival in lysogeny. Another reason could be due to the carrying capacity of the population. If a virulent phage enters a population before their hosts reach a sufficiently high concentration, they may negatively regulate their own resource and thus only reach relatively low concentrations at first. If this is the case, this could hint at a fundamental ecological pattern where temperate phages are more fit during early colonization of an environment while virulent phages have an inherent disadvantage. Quantifying VLP absolute abundances is therefore important for ecological studies of community dynamics, especially in the infant gut microbiome assembly as relative abundances can be misleading.

For example, recent study reported that temperate phages are at higher relative abundance in cesarian born infants compared to vaginal delivered ones^11^. This could equally be explained by the abundant virulent phages from the mother not being able to colonize the infant gut. Another option is that virulent phages simply do not exist for hosts that are abundant in the infant gut. Indeed, it is known that virulent *Bifidobacteria* phages are difficult to isolate *in vitro* and only a handful of isolates exists. On the other hand, in our dataset about half of the *Bifidobacteria* phages are predicted to be virulent and their abundances remained relatively stable over. Thus, the drop in virulent phage abundance in infancy cannot be explained by the abundance of *Bifidobacteria* phages in our dataset.

We observed small and temporary differences in temperate VLP concentration between mothers and infants, suggesting that temperate phage concentrations are comparable between infants and adults. We also found comparable prophage induction (measured by sequencing read coverage) between mothers and infants. Together these data suggests that temperate phage VLPs are maintained by low frequency of spontaneous induction and low amount of lytic replication in the infant gut similar to adults^67,76^. This interpretation is contradicted by one study where induction was estimated to be higher in infants than adults, although there was no statistical test to verify this^77^. However, since prophages can have very specific induction signals, a more granular inspection may reveal individual phage clades that are induced during initial colonization conditions^78^. More research with different cohorts, controlled studies of microbiome transfer and lysogen culturing is needed to confirm these hypotheses.

The majority of vOTUs that were shared between mothers and infants were predicted to infect *Bacteroidaceae*, suggesting that these phages are commonly vertically transmitted from mothers. Indeed, this is expected given that these bacteria and phages have often been suggested to be co-inherited^14,15,18,20,79^. Maternally shared virulent phages were also associated with increased viral load in infants, suggesting that maternal inheritance of virulent phages is important for the maturation of the infant virome. The association became more pronounced during weaning, suggesting that diet interacts with the virome development. Specifically, solid foods include substrates that benefit taxa, such as *Bacteroidaceae*, that can utilize diverse diet derived substrates.

Temperate phages were less frequently shared between mothers and infants than virulent phages despite that they were at higher relative abundances in infants right after birth. This suggests that temperate phages are not commonly inherited from mothers. Indeed, a previous study found that out of 26 vertically transmitted viruses, 21 were predicted to be virulent^9^. The fact that few temperate phages are shared and that there is a negative association with concentration could reflect that when there is inheritance, these phages are more likely to become stable prophages compared to those that are not inherited from mothers. Such phages would include those that were found to be very common in infant metagenomes and increase in prevalence over time like *Lachnospiraceae* and *Bifidobacteriaceae*. Indeed, a recent publication found that prophages in the genus *Bifidobacterium* and *Blautia* are among the bacterial genera with highest proportions of induction in one year old infants^10^. Since the *Bifidobacteriaceae* and *Lachnospiraceae* PHFs do not seem to be from maternal faecal samples, these phage groups may be acquired mainly from other sources such as breastmilk or siblings^14,15,23,80^. More transmission studies are necessary to understand where these phages originate from.

To our knowledge, this is the first study to link faecal metabolite profiles to bacteriophages in the healthy infant gut. The inverse association between virulent phage abundance and primary bile acids is intriguing since bile acids are involved in hormonal, antimicrobial and immunological processes^28,29^. Our data suggests that this relationship may be driven by bacterial hosts involved in or affected by bile acid metabolism rather than direct phage-mediated effects. This is supported by *B. longum* BSH gene abundance having inverse association with bile acids and mediation analysis indicating that the observed phage-bile acid associations of *Bifidobacteriaceae* and *Clostridiaceae* were explained by bacterial hosts. As *Bifidobacterium* species are known to encode BSH proteins that deconjugate primary bile acids and channel them into downstream metabolic pathways^81–84^, the negative association between virulent phages and primary bile acids may reflect phages tracking fluctuations in bacterial populations. However, alternative explanations cannot be excluded. Bile acids are known to influence bacterial growth, phage replication and phage-host interactions^28,85–87^, raising the possibility that elevated bile acid concentrations may directly or indirectly suppress total virulent phage propagation. Conversely, previous experimental work has demonstrated that administration virulent phages can alter bile acid profiles *in vivo*, including reductions of taurochenodeoxycholate 7-sulphate in mice^4^, suggesting that some phages may contribute to shaping bile acid profiles. Thus, although our analysis suggests a host-mediated explanation, the directionality and mechanisms underlying phage-bile acid associations remain unresolved. These findings highlight a connection between phages and bile acid metabolism, emphasizing the need for mechanistic studies that can disentangle direct phage effects from indirect host-mediated responses.

Finally, we found evidence that virulent phage relative abundance followed a bimodal distribution and that functions encoded by mobile genetic elements (MGEs) were more abundant in samples with less virulent phages. This could be either a reflection of more induction occurring in these infants or there being more lysogens, which may be difficult to ascertain. While interpretations should be taken with caution, it could be that infants are colonized by either prophages or virulent phages in a stochastic manner. Another alternative is that temperate phages resist the colonization of virulent phages by encoding defense systems more often. Indeed, temperate phages, prophages and adjacent regions on bacterial chromosomes are known to encode defense systems^25,26^. However, we found no correlation with the virulent phage content and bulk metagenome defense system richness. Discovery of new defense systems and improvement of related bioinformatics could help resolve phage competition and colonization dynamics from metagenomes.

These results highlight maternally derived virulent phages as previously underappreciated players of the early gut ecosystem, increasing our insight into potential roles of phages in the infant gut microbiome. Ultimately, understanding how these inherited viral populations overcome or coexist with resident lysogens and influence metabolite profiles provides a new framework in understanding how phages impact microbiome assembly. Further exploring early-life viral dynamics in relation to different lifestyles and virus-bacterial-host interactions and figuring out how they impact the stool metabolome could provide new strategies to guide the assembly of a healthy gut microbiome during the most critical window of human development.

## Supplementary Material

1

Supplementary Files

This is a list of supplementary files associated with this preprint. Click to download.

- Paper1TableS1.xlsx
- Paper1TableS2.xlsx
- Paper1TableS3.xlsx
- Paper1TableS4.xlsx
- Paper1tableS5.xlsx

## Figures and Tables

**Figure 1.**
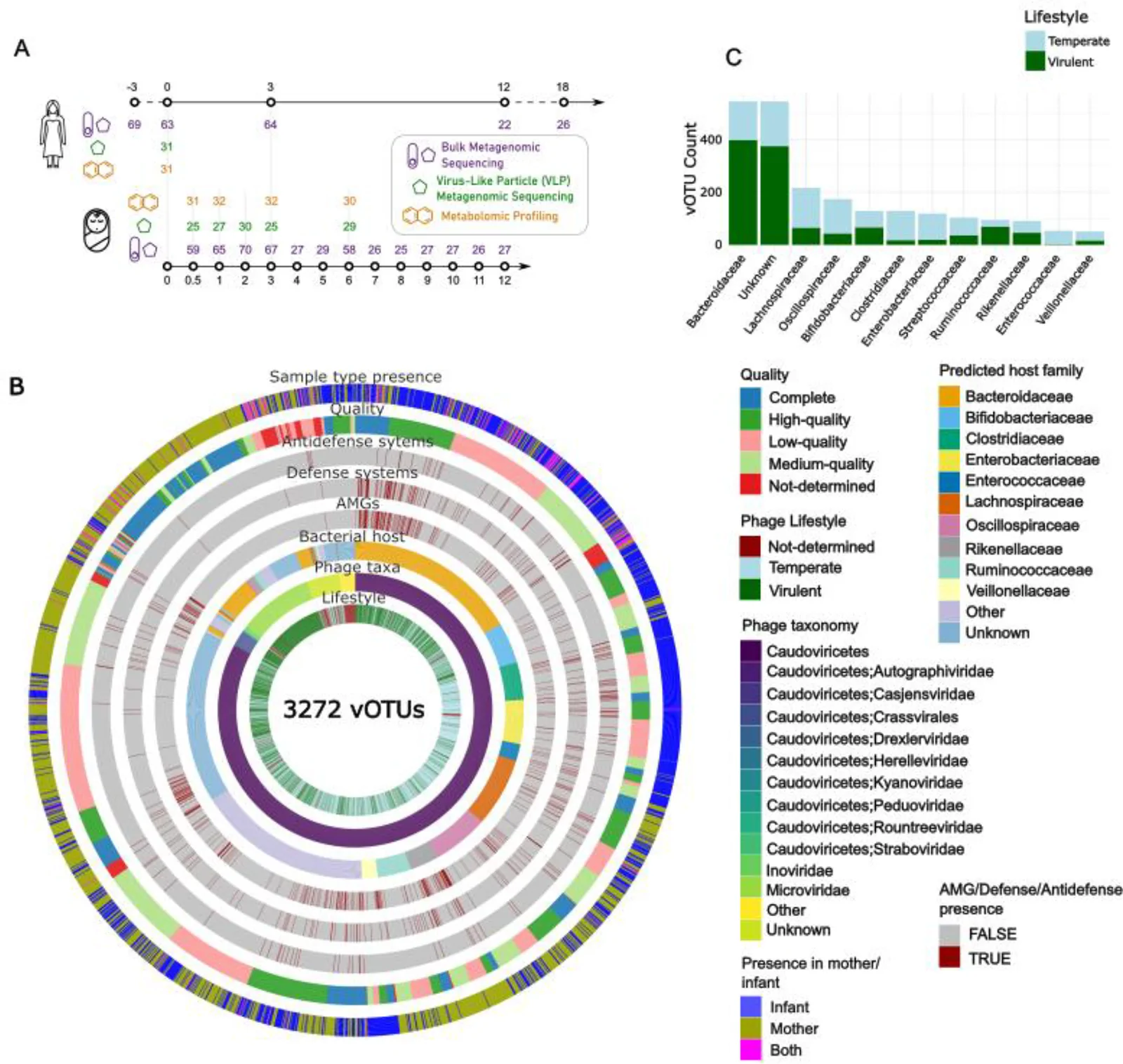
Cohort information and viral Operational Taxonomic Unit (vOTU) characterization. **A.** Types of samples obtained from mothers and infants at different timepoints. Timepoints are displayed in black numbers at the top and bottom and refer to maternal samples taken relative to delivery and infant samples taken at different ages (in months) respectively. Numbers in purple and green represent the amount of bulk and VLP-enriched metagenome samples obtained from feces at each timepoint respectively while numbers on orange represent the number of stool samples where metabolomic profiling was done. **B.** The ringplot represents the characteristics of the 3272 representative vOTUs found in our samples by mapping VLP reads to our catalogue. The ring layers represent a different annotation the vOTU was given and has eight separate layers. Lifestyle: Indicates if prediction software annotated vOTU as virulent, temperate or unknown. Phage taxa: Indicates phage taxonomical classification at class and family level. Bacterial host: Indicates the bacterial host family that the vOTU is predicted to infect. AMGs, Defense systems and Antidefense systems: Indicate if the representative vOTU encodes at least one Auxiliary metabolic gene (AMG), defense system or antidefense system. Quality: Indicates the predicted quality of the vOTU based on checkv categories. Sample type presence: Indicates if the vOTU was present in an infant sample, maternal sample or both. The ring plot is arranged primarily by phage taxa, secondarily by bacterial host and finally by quality. **C.** Stacked barplot showing the number of Caudoviricetes vOTUs predicted to infect different bacterial hosts stratified by lifestyle. The green color represents the number of virulent phages while light blue represents the number of temperate phages with that PHF.

**Figure 2:**
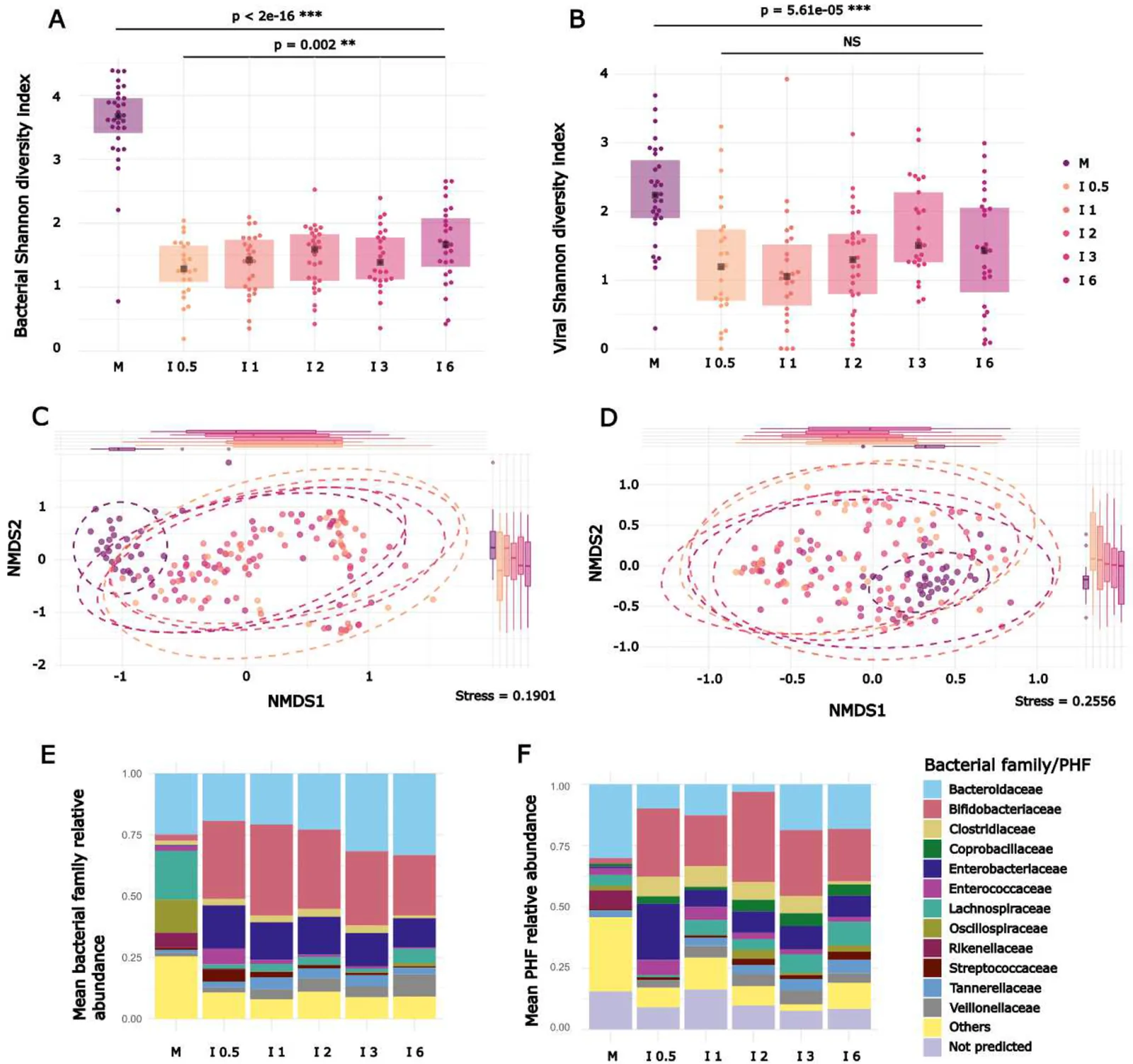
Viral and bacterial diversity and population structures in mothers and their infants. Samples are divided into groups based on whether they are mothers or infants at different ages. M mothers, I 0.5 two-week-old infants, I 1 one month old infants, I 2 two-month-old infants, I 3 three-month-old infants, I 6 six-month-old infants. **A, B**. Boxplots showing bacterial and vOTU Shannon diversity index by group, respectively. Boxplots are colored based on groups and visualize the median and the interquartile range (IQR). Generalized linear mixed effects models demonstrate a significant effect of infant age on bacterial but not vOTU Shannon diversity. Model results are represented by the black lines above the boxplots. Line covering all sample groups represents model comparing Shannon diversity between mothers and infants. Line covering all infant sample groups represents model comparing Shannon diversity change in time. **C, D**. NMDS plots of Bray-Curtis distances of bacterial family and vOTU Predicted Host Family (PHF) abundances respectively. Colors represent the groups each sample belongs to and both NMDS axes have boxplot showing the median, IQR and 1.5 × IQR (whiskers) of these values. Linear mixed models demonstrate that maternal samples and infant age have a significant effect to bacterial family NMDS 1 and PHF NMDS 2. **E, F**. Stacked barplot showing mean relative abundances of bacterial families and PHFs per group. GLMMs show that several bacterial families and their corresponding PHFs have significant positive correlations (Table S2).

**Figure 3.**
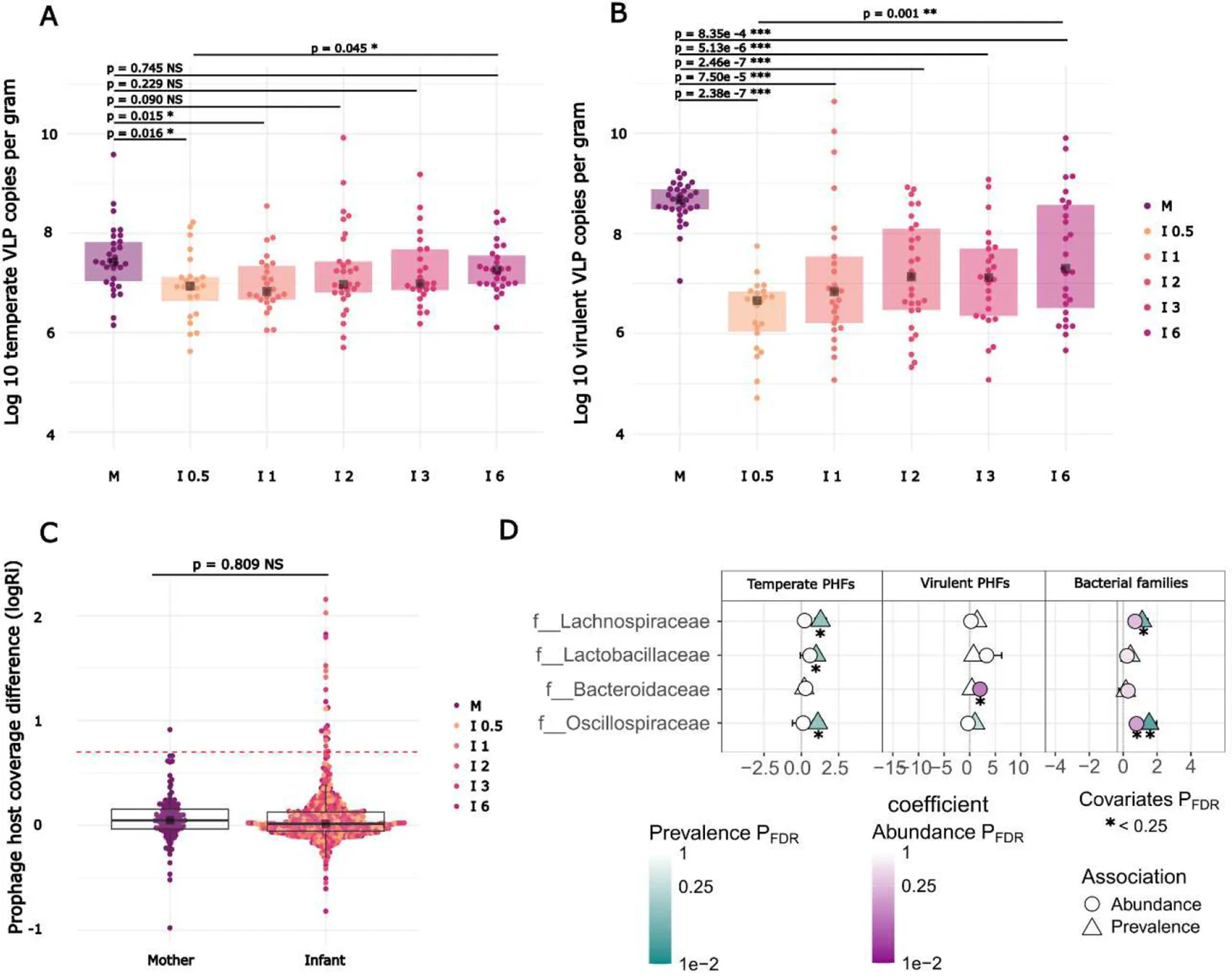
Temperate and virulent phage genome copy concentrations and prophage induction levels in mothers and infants. **A, B**. Estimated fecal temperate and virulent phage concentrations in mothers and in infants over time. Four samples <10^4^ copies per gram were excluded to improve clarity. Boxplots are colored based on sample groups and visualize the median and IQR. Infant age had a statistically significant correlation with the temperate and virulent phage absolute abundances (LMM, p=0.45 for temperate phage, p=0.001 for virulent phage). All infant samples had lower virulent phage abundances and infant samples collected at 0.5 and 1 month of age had lower temperate phage abundances compared to the maternal samples (Paired Wilcoxon signed rank test; *p<0.05, **p<0.01, ***p<0.001). **C**. Sequencing read coverage difference between prophages and their flanking genomic sequence in maternal and infant samples. The dots represent each prophage-contig pair and are colored by maternal and infant sample timepoint it originated from. Boxplot shows median, IQR and IQR × 1.5. The dashed red line represents the threshold coverage difference to be considered significantly induced by the Propagate. There was no significant difference between mothers and infants (LMM, p=0.809). **D**. Correlations between infant age and bacterial or phage abundances and prevalences as determined linear mixed models in MaAsLin3. Three sections visualize the model coefficients (β) of temperate PHF concentrations, virulent PHF concentrations and bacterial family relative abundances. The triangles (green) and circles (purple) represent the coefficients of prevalences and abundances, respectively. The intensity of the color represents the FDR corrected p-value (P_FDR_) of the association, and an asterisk represents values with P_FDR_<0.25.

**Figure 4.**
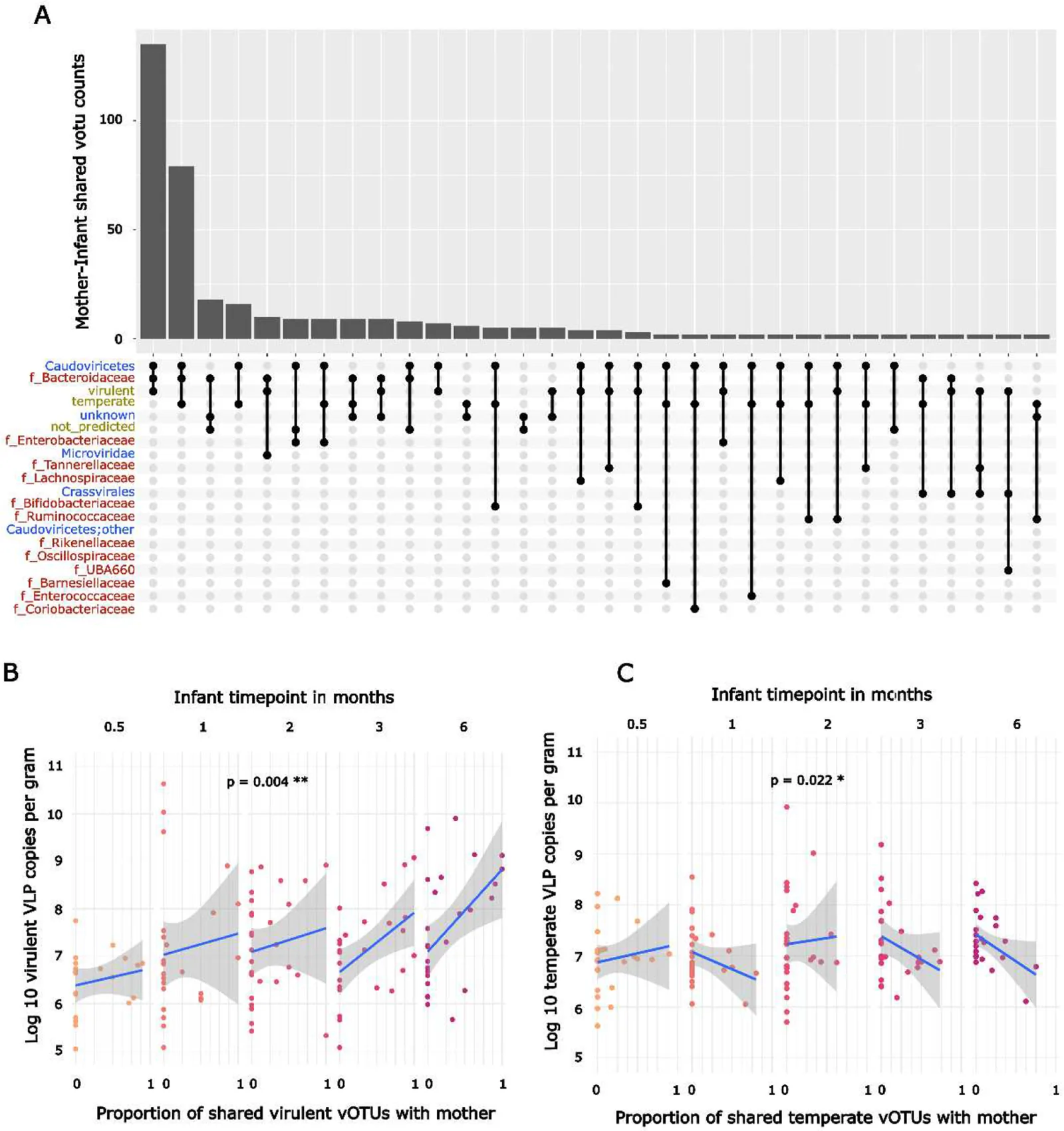
Inherited and persistent vOTUs and their characteristics. **A**. The frequency of vOTUs shared between mothers and infants broken down by phage lifestyle (dark yellow text), PHF (dark red text) and taxonomy (dark blue text). *Bacteroidiaceae* infecting virulent *Caudoviricetes* were the most frequently shared category. **B.** LMM model of the relationship between the proportions of maternal shared virulent vOTUs, virulent vOTU concentrations and infant age. **C.** Visual representation of LMM modelling of the relationship between the proportions of maternal shared temperate vOTUs, temperate vOTU concentrations and infant age. Both visualizations show five scatterplots of proportion of active vOTUs shared with mothers and vOTU genome copies per gram by each infant timepoint separately to show how the relationship changes by the third and sixth months. Each scatterplot shows a line of best fit for each infant timepoint with a 95% confidence interval in gray. The result of the effect of temperate and virulent sharedness on VLP copies according to our LMMs are displayed on each figure.

**Figure 5.**
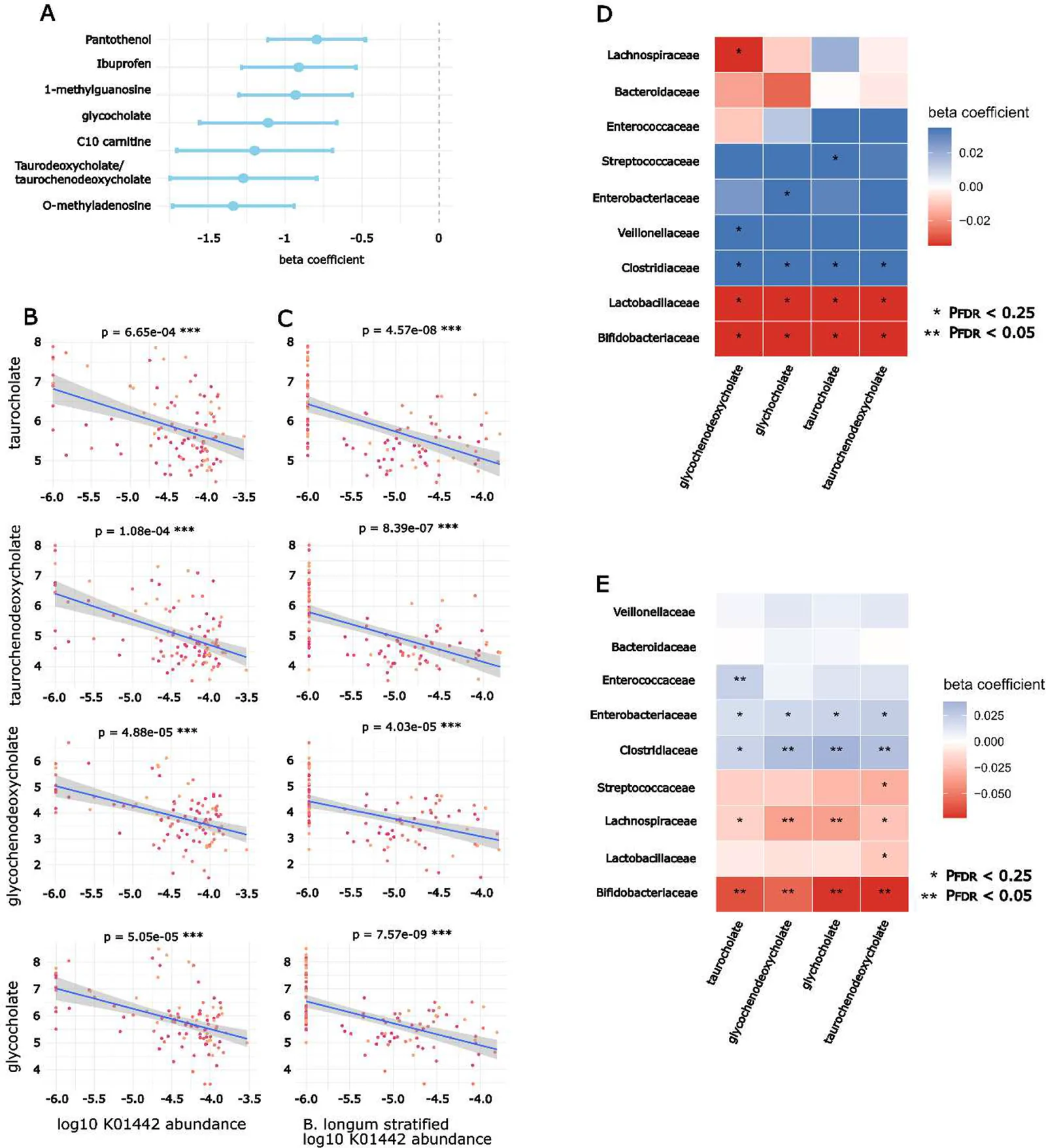
Correlations between fecal metabolites and microbes. **A**. Annotated infant fecal metabolites that correlated with virulent phage concentrations (LMM, P_FDR_<0.25). **B**. Linear models between bile salt hydrolase (BSH, K01442) relative abundance and four primary bile acids concentrations measured by C18-neg LC-MS as modeled by LMMs. The numbers above each graph show the p-value for the effect in each model. **C**. Linear model between *Bifidobacterium longum* BSH relative abundance and four primary bile acids concentrations measured by C18-neg LC-MS as modeled by LMMs. The numbers above each graph show the p-value for the effect in each model. Each scatterplot in **B** and **C** shows a line of best fit with a 95% confidence interval in gray. **D**. Associations between PHF absolute abundance and four primary bile acids concentrations measured by C18-neg LC-MS modeled using LMMs. **E**. Associations between relative abundance of bacterial families and four primary bile acids concentrations measured by C18-neg LC-MS modeled using LMMs. Heatmaps in **D** and **E** represent the beta coefficients from models controlling for infant age, exclusive breastfeeding and gender.

**Figure 6.**
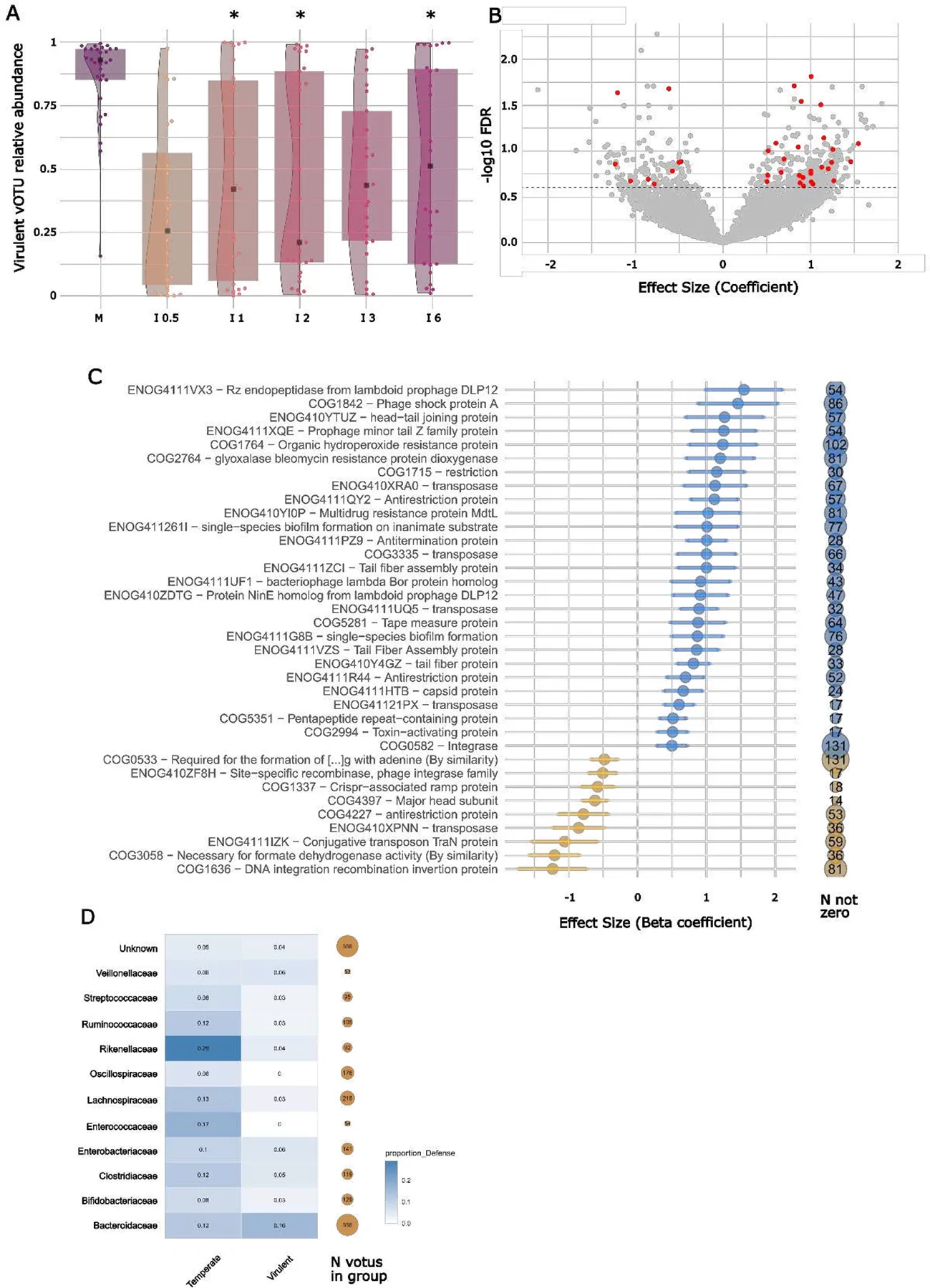
Stratifying viral communities by relative abundance of virulent phages. **A.** Relative abundances of virulent phages in each time point. Boxplots are colored based on time points and the median and IQR. Violin plot shows the distribution of relative abundances in each group and the asterisk above each time point refers to the group having a bimodal distribution (Hartigans’ dip test, P<0.05). **B**. Volcano plot of associations between abundance of HUMAnN3 estimated COG and NOG abundances and virulence groups (high/low). The black horizontal line indicates P_FDR_< 0.25 and the red dots indicate terms related to mobile genetic elements and phage defense with P_FDR_<0.25. **C**. COG and ENOG terms related to mobile genetic elements and phage defense with abundance differences between virulence groups (red points in panel **B**). The plot shows each COG and ENOG description, model coefficient (β) and the number of metagenomes encoding function. **D**. Phage defence system carriage stratified by phage lifestyles and PHFs. The heatmap color intensity indicates prevalence between vOTUs. The size of the yellow circles on the right hand side indicate to the number of vOTUs per PHF.

## Data Availability

Processed VLP enriched metagenome reads have been deposited to NCBI Sequence Read Archive under BioProject PRJNA821542. MIUViG sequences from table S1 and vOTU catalogue sequences will be published online upon article publication. Relevant code used for analysis can be found in github (https://github.com/KimPhage/EDIA_virome).
