# Supplementary material for: Maternal transmission and limited virulent phage replication shape early infant gut virome maturation
Source: Res Sq. 2026 Oct 5:rs.3.rs-11232673. Preprint. [Version 1] doi: 10.21203/rs.3.rs-11232673/v1 (PMC13692616; doi:10.21203/rs.3.rs-11232673/v1)
Supplement: Supplementary file 1 [file NIHPPRS11232673V1-supplement-1.pdf]

Supplementary information

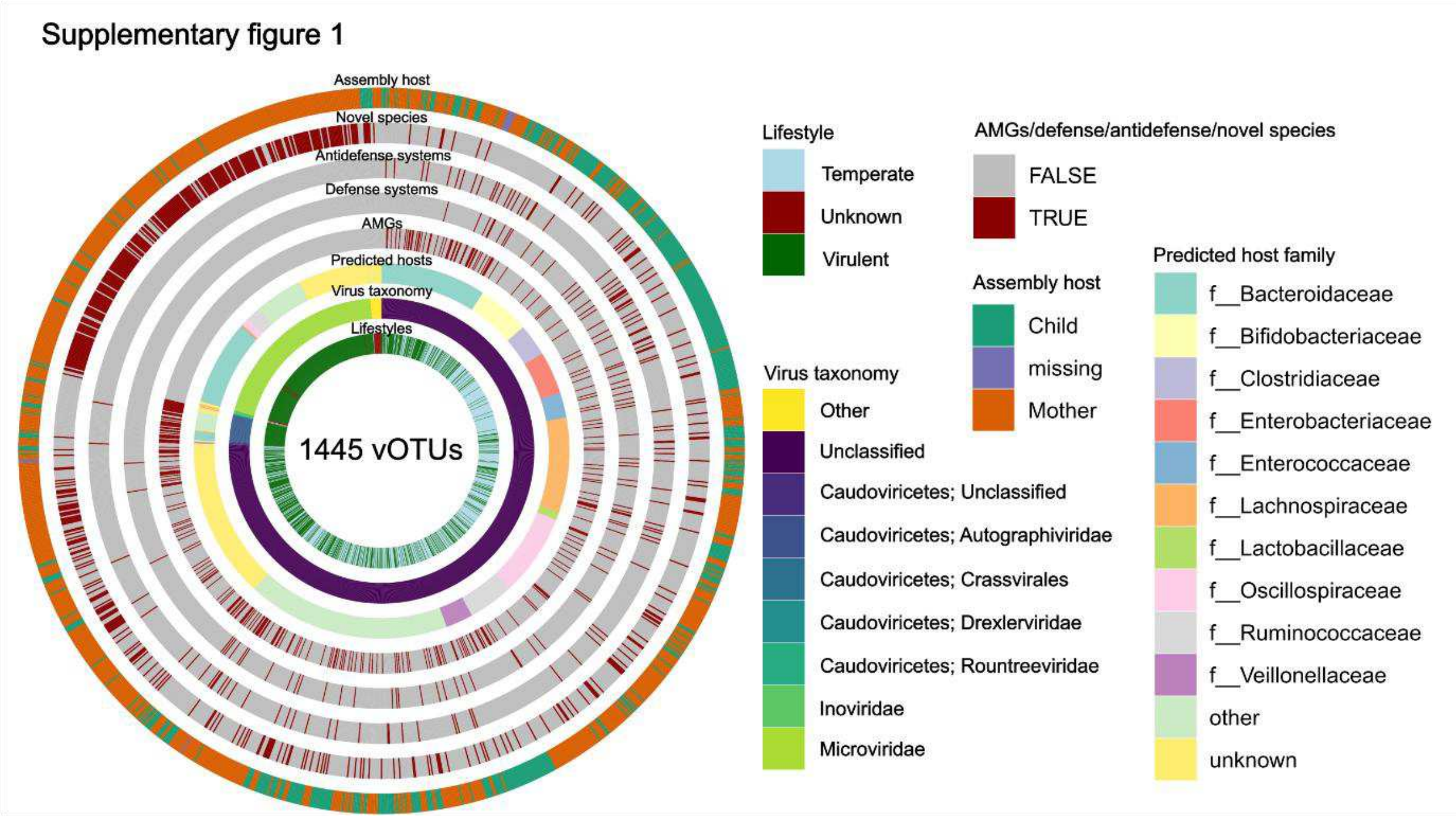

Supplementary Figure 1. Assembled representative quality vOTUs. Ringplot represents the characteristics of the 1445 medium, high and complete quality vOTUs *de novo* assembled from VLP and bulk metagenome reads and clustered to 95% average nucleotide identity (ANI) by 85 % coverage. The ring layers represent a different annotation the vOTU was given and has eight separate layers. Lifestyle: Indicates if prediction software annotated vOTU as virulent, temperate or unknown. Phage taxa: Indicates phage taxonomical classification in class and family level. Bacterial host: Indicates the bacterial host family that the vOTU is predicted to infect. AMGs, Defense systems and Antidefense systems: Indicate if the representative vOTU encodes at least one AMG, defense system or antidefense system. Novel species: Indicates if the assembled vOTU clustered with vOTUs from other databases or not. Assembly host: Indicates what sample the vOTU was assembled from.

Supplementary figure 2

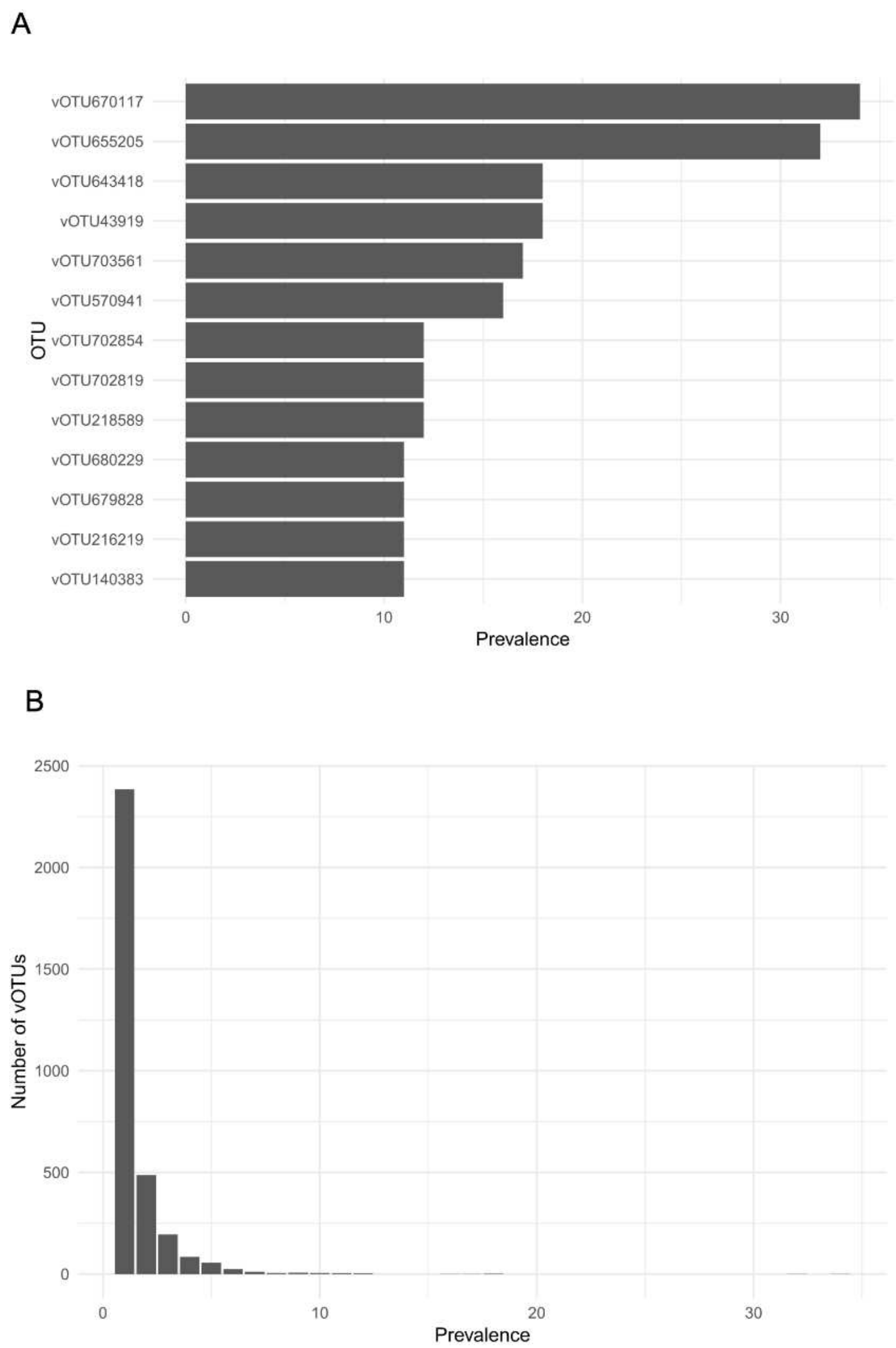

Supplementary Figure 2. vOTU prevalence statistics. A. Bar plot shows the top 13 most prevalent vOTUs in the cohort and how many samples each of the vOTUs was found based on VLP read mapping. **B.** Histogram showing the frequency of vOTUs found in one sample or more.

Supplementary figure 3

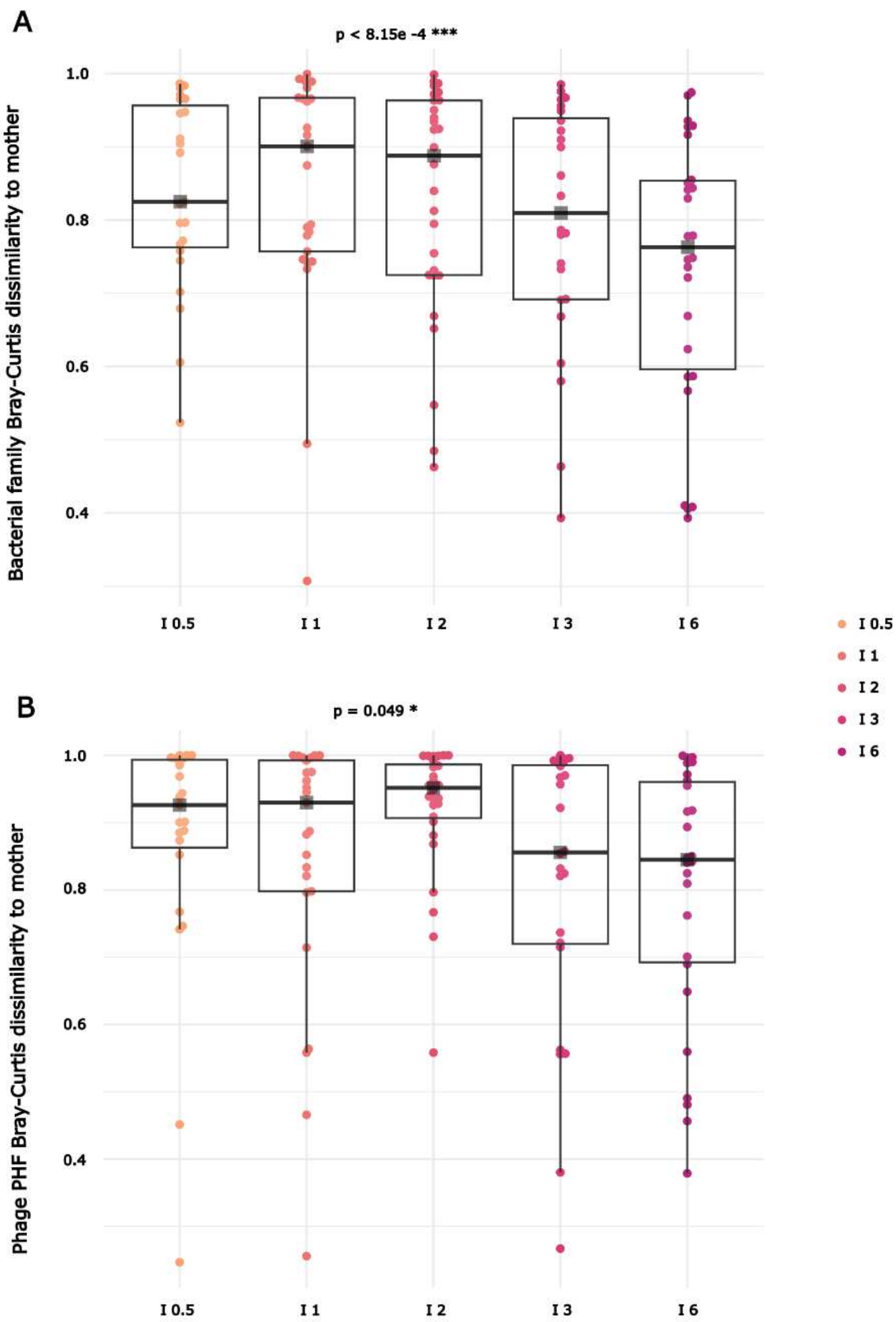

Supplementary Figure 3. Bacterial and phage community similarity between mother-infant pairs across infant age. A. Bar plot shows the bacterial family Bray-Curtis dissimilarity between infants at each timepoint and their mothers. **B.** Bar plot shows the PHF Bray-Curtis dissimilarity between infants at each timepoint and their mothers. The statistics above each plot represent the effect of infant age on the change in dissimilarity according to the LMMs. Boxplots show the median, IQR and IQR x 1.5.

Supplementary figure 4

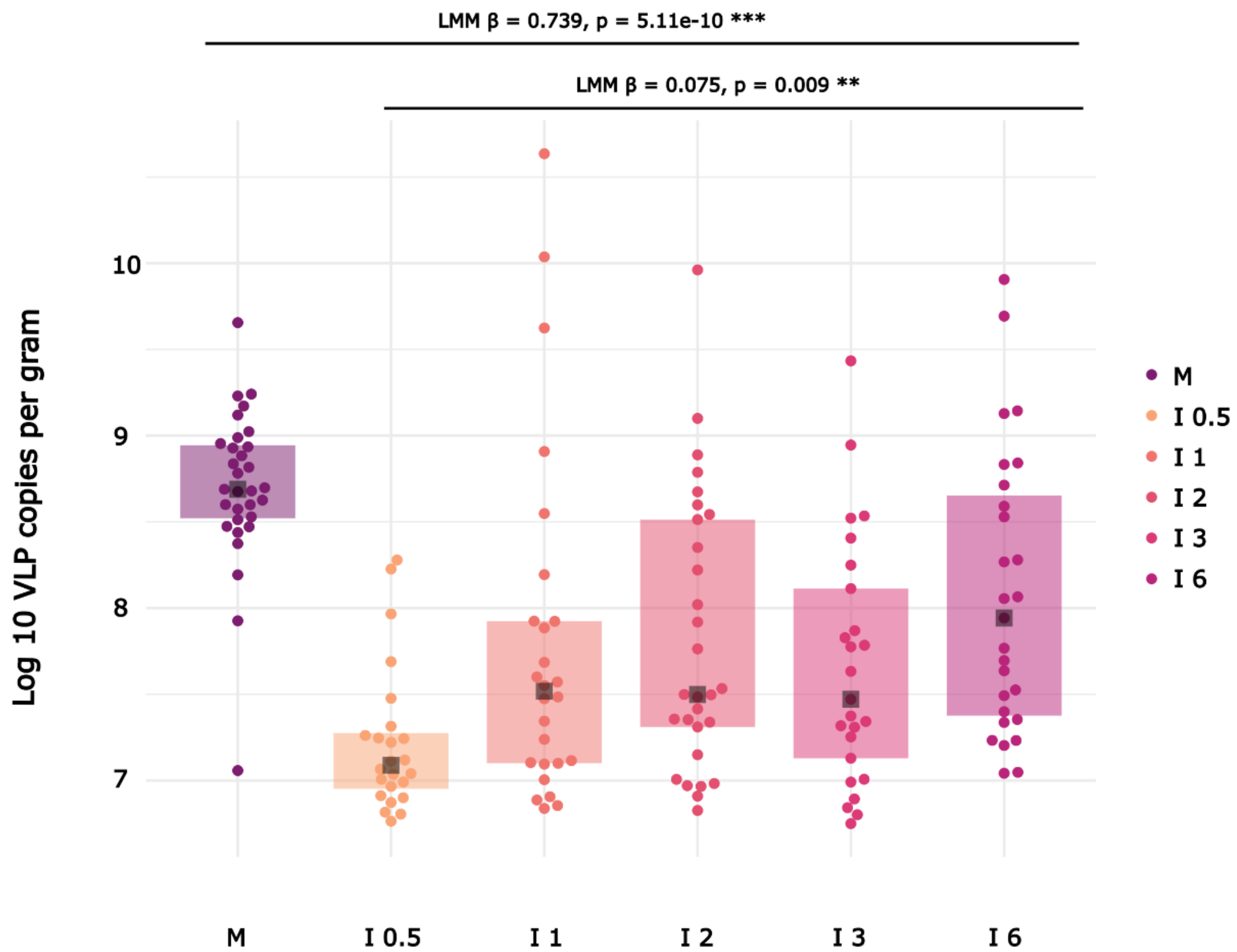

Supplementary Figure 4. VLP concentration across samples. Boxplot showing the estimated VLP genome copies per gram of feces in log^{10} scale for different groups. LMMs show there is a significant increase in VLP genome copy concentrations with infant age and that there is a significant difference between VLP concentrations between mothers and infants. Boxplots are colored based on sample groups and show the median and IQR. LMM results are represented by the black lines above the boxplots. Line covering all sample groups represents model comparing VLP genome copy concentrations between mothers and infants. Line covering all infant sample groups represents model comparing VLP genome copy concentration change in time.

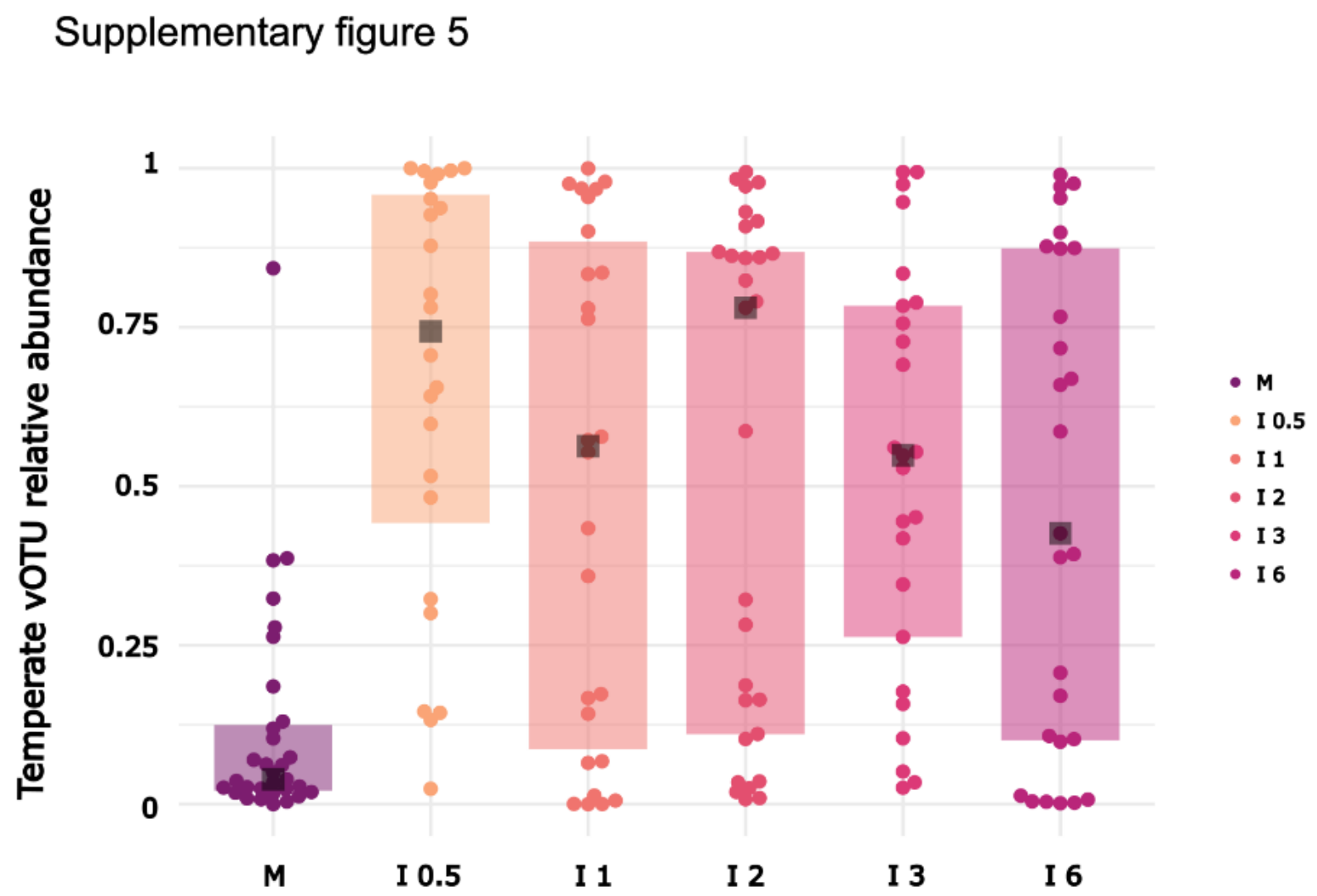

Supplementary Figure 5. Viral lifestyle relative abundance across samples. Boxplot showing the relative abundance of active temperate phages in different groups. Boxplots are colored based on sample groups and show the median and IQR.

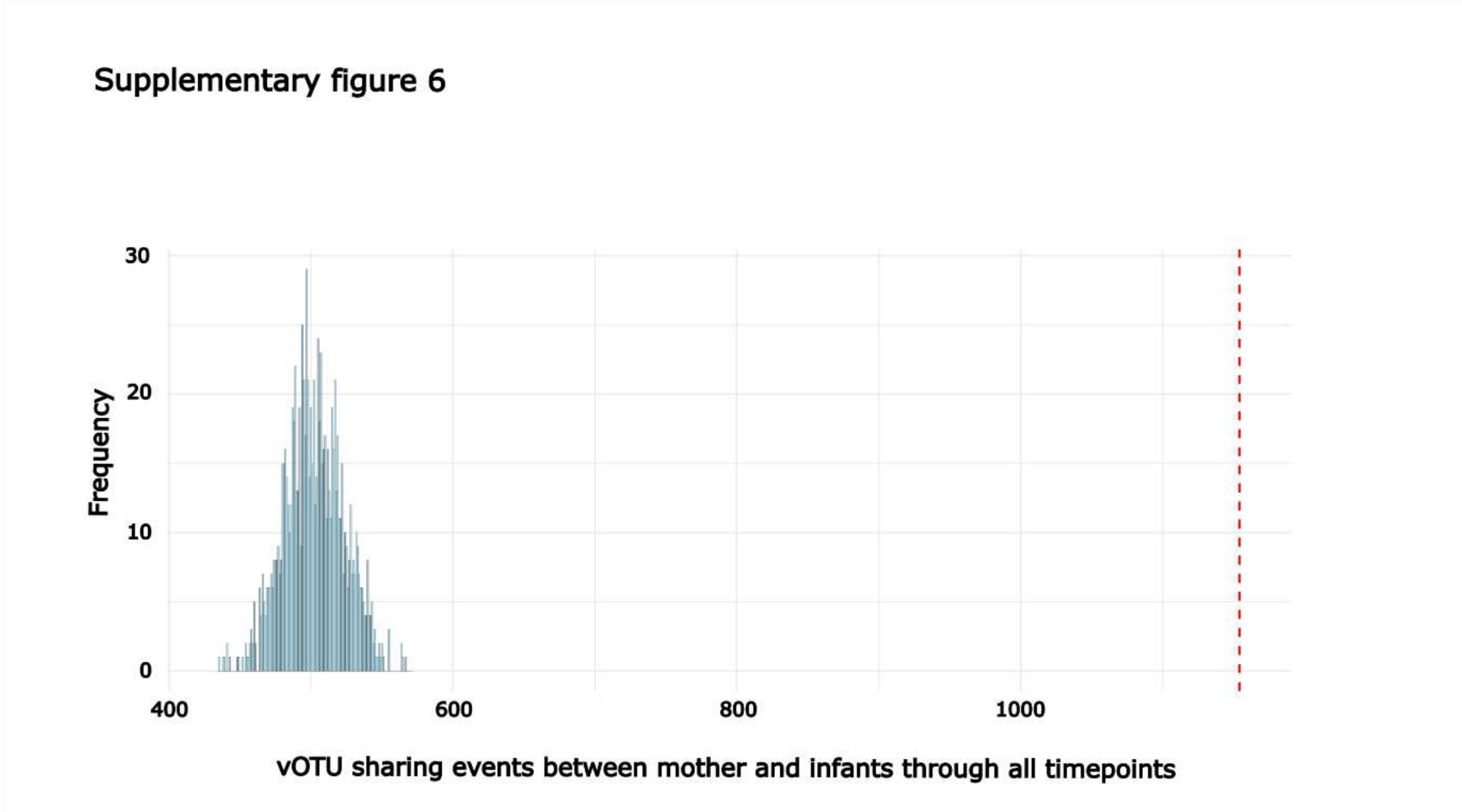

Supplementary Figure 6. Mother-Infant shared vOTU permutation analysis. Histogram shows results of the permutation analysis with 1000 replications. Histogram in blue depicts the frequency of vOTUs being shared between mother and their child if they were randomly shuffled. The red line is the observed amount of vOTU sharing events and is significantly different from the permuted values ($P<0.001$).

Supplementary figure 7

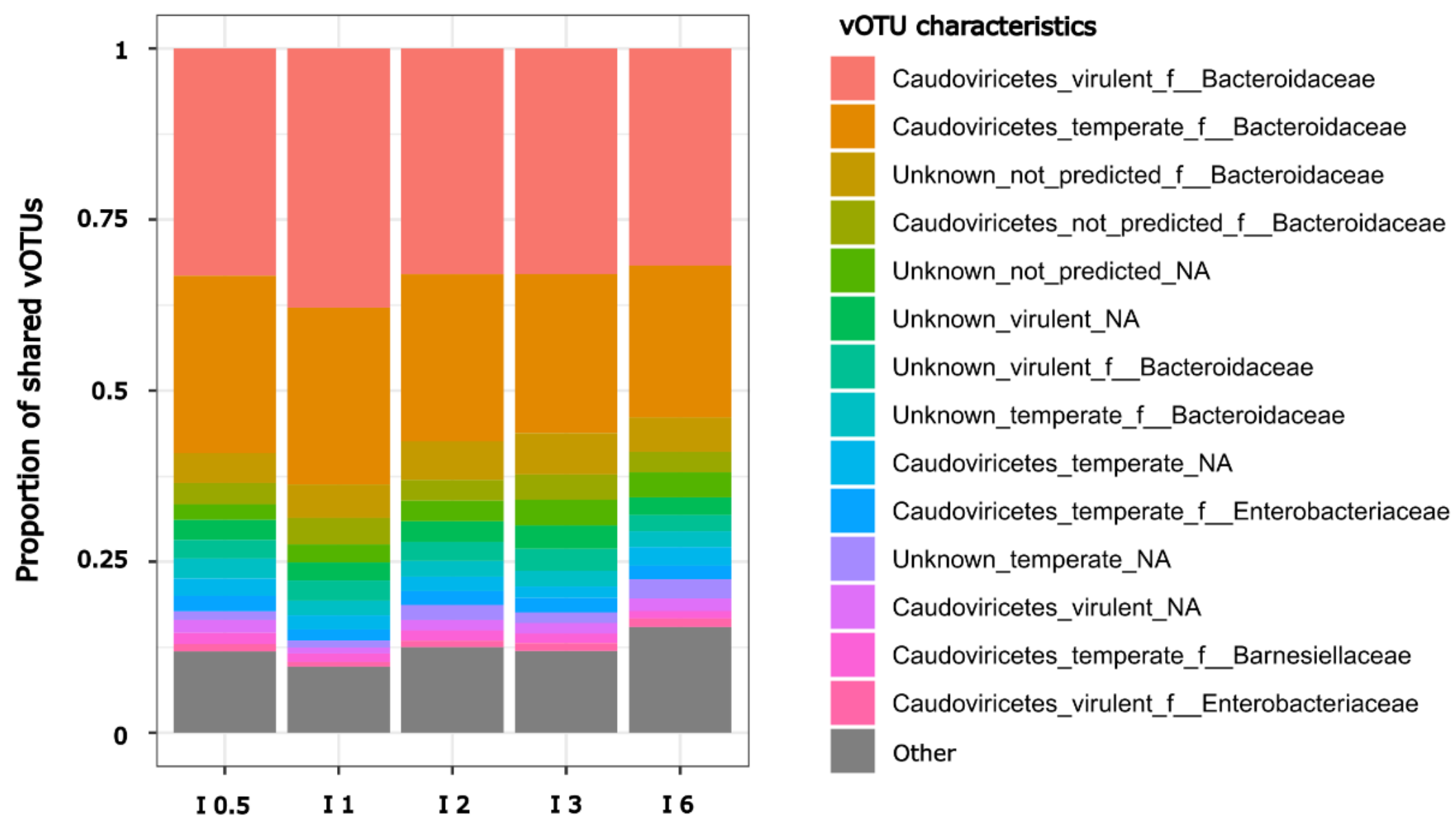

Supplementary Figure 7. Composition of mother-infant shared vOTUs across infant age. Stacked barplot showing the proportion of grouped vOTU shared with mothers at each timepoint. The vOTUs were grouped by their predicted taxonomies, lifestyle and PHF (same as Fig 4A). The top fourteen most shared groups are shown, and the rest are grouped as other.

Supplementary tables

Table S1: Collection of the MiUVIG metadata of the 1445 representative vOTUs assembled from this cohort.

Table S2: GLMM results of bacterial family and PHF correlations.

Table S3: P_{FDR} filtered LMM results of fecal metabolites affected by phage, virulent phage and temperate phage VLP concentrations.

Table S4: Mediation analyses results of phage, bacteria and primary bile acid LMMs for Clostridiaceae and Bifidobacteriaceae families and PHFs.

Table S5: P_{FDR} filtered LMM results of bacterial SGBs affected by infant age, virulent phage content and exclusive breastfeeding covariates.
